# Genomic and transcriptional analysis of genes containing fibrinogen and IgSF domains in the schistosome vector *Biomphalaria glabrata*, with emphasis on the differential responses of snails susceptible or resistant to *Schistosoma mansoni*

**DOI:** 10.1371/journal.pntd.0008780

**Published:** 2020-10-14

**Authors:** Lijun Lu, Eric S. Loker, Coen M. Adema, Si-Ming Zhang, Lijing Bu

**Affiliations:** Center for Evolutionary and Theoretical Immunology (CETI), Department of Biology, University of New Mexico, Albuquerque, New Mexico, United States of America; University of the District of Columbia, George Washington University School of Medicine and Health Sciences, UNITED STATES

## Abstract

Achieving a deeper understanding of the factors controlling the defense responses of invertebrate vectors to the human-infecting pathogens they transmit will provide needed new leads to pursue for control. Consequently, we provide new genomic and transcriptomic insights regarding FReDs (containing a fibrinogen domain) and FREPs (fibrinogen domain and one or two IgSF domains) from the planorbid snail *Biomphalaria glabrata*, a Neotropical vector of *Schistosoma mansoni*, causative agent of human intestinal schistosomiasis. Using new bioinformatics approaches to improve annotation applied to both genome and RNA-Seq data, we identify 73 FReD genes, 39 of which are FREPs. We provide details of domain structure and consider relationships and homologies of *B*. *glabrata* FBG and IgSF domains. We note that schistosome-resistant (BS-90) snails mount complex FREP responses following exposure to *S*. *mansoni* infection whereas schistosome-susceptible (M line) snails do not. We also identify several coding differences between BS-90 and M line snails in three FREPs (2, 3.1 and 3.2) repeatedly implicated in other studies of anti-schistosome responses. In combination with other results, our study provides a strong impetus to pursue particular FREPs (2, 3.1, 3.2 and 4) as candidate resistance factors to be considered more broadly with respect to schistosome control efforts, including involving other *Biomphalaria* species vectoring *S*. *mansoni* in endemic areas in Africa.

## Author summary

Just as malaria parasites depend on mosquitoes for transmission, *Schistosoma* trematodes that infect 200+ million people in developing countries depend on freshwater snails to support their development. New control methods targeting schistosomes in the snail host are urgently needed to help break the cycle of transmission. Identification of molecules in snails that confer or facilitate resistance to successful larval development offers one way forward. Building on previous studies and new data, we used a combination of bioinformatics tools to characterize a repertoire of molecules in *B*. *glabrata* containing fibrinogen or IgSF domains, or both (the latter molecules called FREPs, first discovered in *B*. *glabrata*). We found that snails resistant to schistosomes mount vigorous, multi-component FREP responses following exposure to schistosomes whereas responses of susceptible snails are modest by comparison. Furthermore, for four FREPs repeatedly implicated in snail responses to trematodes, three (FREPs 2, 3.1 and 3.2) exist in distinctive allelic forms in susceptible and resistant snails. We propose that the search for homologous molecules needs to move to the field, to *B*. *glabrata* in the Neotropics and to other vector species of *Biomphalaria* in Africa, to explore the potential of FREPs as resistance factors worthy of development as control agents.

## Introduction

Human schistosomiasis, one of the most persistent and common of the neglected tropical diseases, is caused by digenetic trematodes in the genus *Schistosoma* [1]. *Schistosoma mansoni* causes intestinal schistosomiasis and infects 83 million [2] of the more than 200 million people in the world estimated to need protective treatment from the disease annually [3,4]. Transmission of *S*. *mansoni* to people depends on the presence of compatible species of the freshwater planorbid snail genus *Biomphalaria* which serve as obligatory intermediate hosts. Snails become infected when penetrated by miracidia hatched from *S*. *mansoni* eggs passed in human feces. Within infected snails schistosome sporocysts eventually produce thousands of cercariae which leave the snail body, contact and penetrate human skin and develop into adult worms. The most important snail host for *S*. *mansoni* in the Neotropics is *B*. *glabrata*. The combination of *S*. *mansoni* and *B*. *glabrata* has become an oft-studied model for trematode-snail interactions and for both molluscan and invertebrate immune response capabilities [5,6,15–17,7–14].

**Fig 1:**
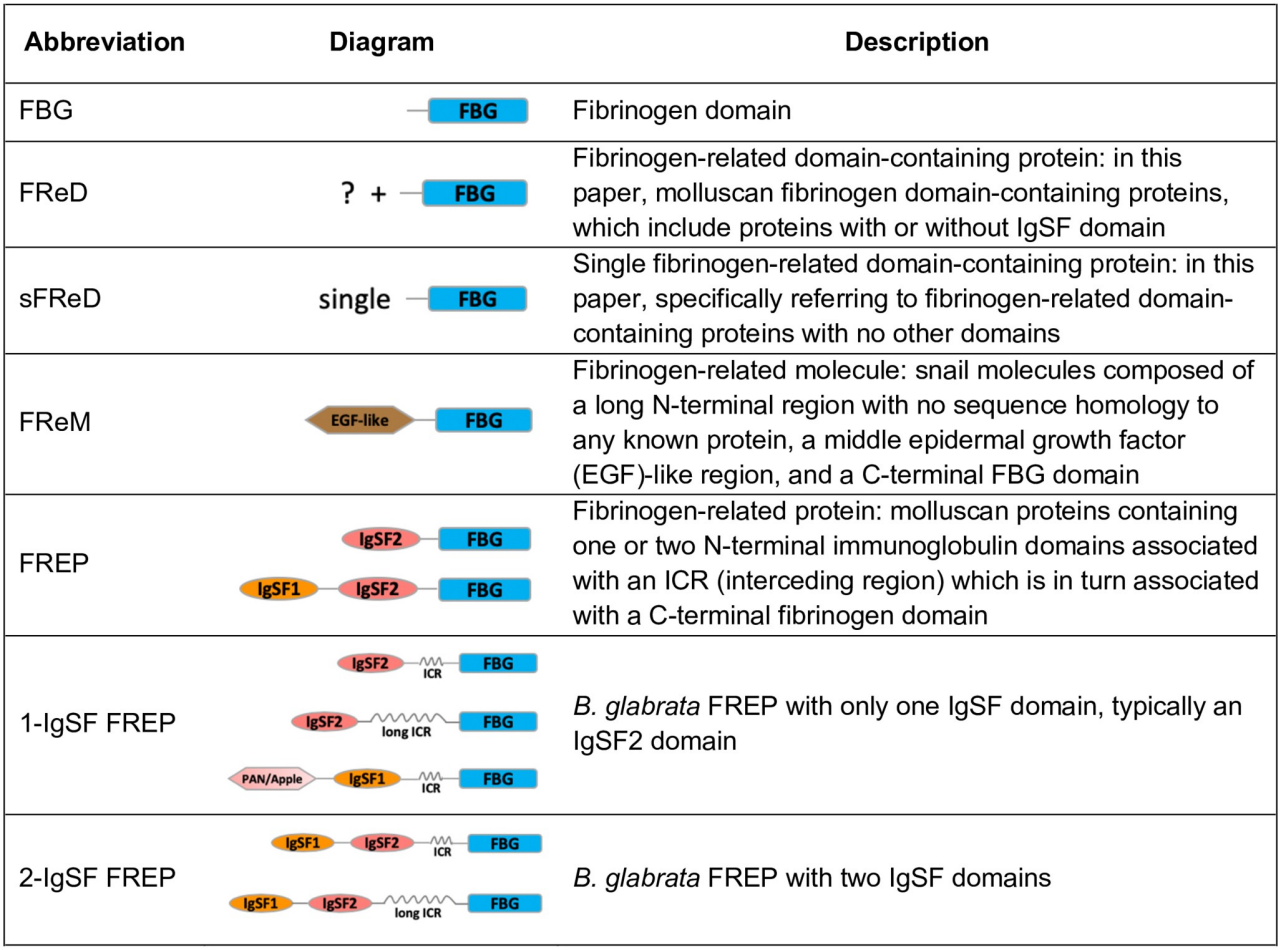
Some relevant abbreviations used in this paper. Detailed domain sturcture for each abbreviation can be found in Fig 3. FBG: fibrinogen domain; EGF-like: epidermal growth factor (EGF)-like domain; IgSF1: Immunoglobulin Superfamily Member 1; IgSF2: Immunoglobulin Superfamily Member 2; ICR: interceding region (<150aa) between IgSF and FBG domains that form coiled coils; long ICR: long interceding region (150~320aa).

Our goal in this paper is to provide a comprehensive genomics-based overview, along with insights from their transcriptional profiles, for a group of defense-related molecules from *B*. *glabrata* known as fibrinogen-related domain containing proteins, or FReDs (Fig 1). In addition to the fibrinogen-related (FBG) domain, FReDs may or may not also contain IgSF domains. Among the FReDs from *B*. *glabrata* is a family of diverse hemolymph lectins [18,19] originally termed fibrinogen-related proteins, or FREPs [20]. FREPs have become one of the most studied groups of invertebrate defense molecules and for this reason are a particular focus of this paper. FREPs contain one or two N-terminal immunoglobulin superfamily (IgSF) domains, an interceding region (ICR without distinctive homology to other domains) and a C-terminal FBG-like domain (Fig 1). IgSF members, which share a conserved immunoglobulin domain or fold but exhibit a wide range of amino acid sequence variation, are well known for their role of ligand binding, showing high specificity and diverse recognition capacity [21]. In animals, IgSF domain-containing proteins are involved in cell-cell recognition, cell-surface receptors, muscle structure and the immune system [22,23]. Invertebrate IgSF domain-containing proteins are also capable of binding to various pathogen-associated molecular patterns [20,24–26]. IgSF domains share a common Greek key-like core with beta-sandwiches. Based on differing numbers of beta-sandwiches, IgSF domains are grouped into four types: V-set (variable), C1-set (constant-1), C2-set and I-set (intermediate) [27,28]. FREP-associated IgSF1 and IgSF2 domains both resemble V-type IgSF domains based on sequence similarity and immunoglobin loop size [26,29], but they are readily separable from one another with respect to their amino acid sequences, designated as IgSF1 and 2 [26]. In 1-IgSF FREPs, it is the IgSF2 domain that is present, and in 2-IgSF FREPs, the IgSF1 is N-terminal and the IgSF2 domain lies immediately upstream of the ICR and the relatively conserved C-terminal FBG domain [26,29–31].

Starting with an initial characterization of 14 *B*. *glabrata* FREPs [29,30], the genome-based study of *B*. *glabrata* BB02 strain went on to reveal 22 FREP genes, 19 with two tandem IgSF domains and 3 with one IgSF domain [32]. In a study of the *de novo* assembled transcriptome of the BgBRE strain of *B*. *glabrata*, 28 transcripts covered at least IgSF and FBG domains, yielding 8 new FREPs [33], bringing the known total to 30. Also, in an RNA-Seq study of multiple strains of *B*. *glabrata*, a FREP-specific transcriptome consisting of 69 transcripts was assembled, and 24 genomic loci potentially encoding for 69 complete or partial FREP transcripts were identified [34], bringing the known number of distinct FREP loci to 32. One of the most distinctive features of FREP biology is, for a particular FREP gene such as *BgFREP3*.*2*, to recover variant forms of genomic DNA and mRNA from different cells (such as from the circulating defense cells called hemocytes) from a single snail. These variants derive from characteristic germ-line source sequences through both gene conversion and point mutation processes [35], providing additional FREP diversity [31].

FREPs have drawn attention because of their responsiveness upon exposure to relevant pathogens, especially digenetic trematodes of biomedical significance like *S*. *mansoni*. With the exception of the study by Galinier et al. [34], all of these studies pre-date the RNA-Seq era [20,36–44]. As circulating hemolymph lectins, the abundance of FREPs increases in the blood of *B*. *glabrata* following exposure to some trematodes. FREPs are also known to bind to sporocysts in a carbohydrate-inhibitable manner and can act as opsonins to facilitate phagocytosis of particles by hemocytes [35]. Microarray studies have also confirmed that a particular FREP, *FREP3*.*2*, is up-regulated in *B*. *glabrata* known to be resistant to the digenean *S*. *mansoni* [39]. Furthermore, use of RNAi to specifically knock-down FREP3.2 expression diminished the resistance shown to either echinostome or schistosome trematode larvae [38,40].

With respect to arriving at a mechanistic understanding of snail susceptibility or resistance, it is known that *S*. *mansoni* has a large repertoire of genes encoding polymorphic mucin molecules (SmPoMucs) [45]. Given that these are displayed on the surface of sporocysts, it has been argued that particular combinations of expressed FREPs and SmPoMucs act as determinants of compatibility, thus providing an allele vs. allele matching system that can also help to explain the specificity of compatibility [46]. A recent functional study of several humoral immune factors from *B*. *glabrat*a revealed that an association forms between *B*. *glabrata* FREP3.2 (here confirmed as FREP3.2, AAK28656.1), thioester-containing protein 1 (TEP1), biomphalysin and FREP2. It was observed that FREP3.2 can associate with TEP1 and biomphalysin in both schistosome-susceptible M line (SUS) and resistant BS-90 (RES) snails, but only in RES snails does this immune complex uniquely recruit representatives of FREP2 and of FREP3 distinct to RES snails. One version of FREP3 specifically mentioned in this context was FREP3.3, a FREP locus not yet fully assembled in the current version of the genome [47]. From the earliest studies of FREPs to more recent studies on the binding preferences of FREPs [36,47–50], it has been of interest to learn if and how the FREPs encoded by SUS and RES snails might differ. The approach taken in our study allows us to address this issue.

In addition to FREPs, we are interested in other *B*. *glabrata* FReDs. The FBG domain is a conserved component of bioactive molecules in a wide range of taxa [51], and is well-known for its role in coagulation in vertebrates [51]. It is generally considered FBG domains in invertebrates play roles in defense responses [30,52,53]. Invertebrate FBG domains have been discovered in genes involved in 1) recognizing bacterial molecules containing acetyl groups [54–56]; 2) regulating defense-related enzymatic pathways [57,58]; 3) eye and central nervous system development [59–61]; and 4) allorecognition [62,63]. A proteomics study characterizing *B*. *glabrata* hemolymph proteins binding to proteins from *S*. *mansoni* mother sporocysts [49] found that along with FREPs, sFReDs (Fig 1) could also bind parasite molecules, again suggestive of a role in defense.

From many other studies it is also clear that several humoral factors other than FReDs and FREPs deserve additional consideration for the role they may play in influencing the degree of compatibility between schistosomes and their snail hosts [12,16,17,47,64,65]. We in no way wish to deemphasize their potential importance, but for the purposes of this study, our focus is on FReDs and FREPs. In this study, we developed a novel automated method to accurately identify IgSF domains. Together with traditional IgSF and FBG domain identification methods, we provide the first comprehensive genome-wide investigation of *B*. *glabrata* FReDs (including FREPs) structure and function. We explored relationships among *B*. *glabrata* FReDs and depict the diversity of IgSF and FBG domains. Expression profiles of FReDs in both RES and SUS strains of *B*. *glabrata*, with or without *S*. *mansoni* exposure, are documented and related to previous studies relating the role of FREPs in resistance to schistosomes. We also highlight differences in coding regions between key FREP genes between RES and SUS snails.

## Methods

### FBG domain searches with InterProScan

The FBG domains were identified from protein sequences derived from the *B*. *glabrata* BB02 genome reference annotation version 1.6 downloaded from VectorBase (https://www.vectorbase.org/organisms/biomphalaria-glabrata/bb02/bglab16). Initial functional domain searches were performed using InterProScan v5.34–73.0 [66] with databases including TIGRFAM (15.0), SFLD (4), SUPERFAMILY (1.75), PANTHER (14.1), Gene3D (4.2.0), Coils (2.2.1), SMART (7.1), CDD (3.16), PRINTS (42.0), ProSitePatterns (2019_01), Pfam (32.0), ProDom (2006.1) and PIRSF (3.02). Signal peptides were predicted using SignalP 4.1 [67] and trans-membrane domains were predicted using TMHMM 2.0 [68]. Genes containing an FBG domain were identified using a keyword “fibrinogen” search in InterProScan results and confirmed with FBG domain identifications in several different databases of PF00147 (Pfam), SM00186 (SMART), cd00087 (CDD) and SSF56496 (SUPERFAMILY). Domain prediction results with overlapping regions from different programs or databases were resolved based on *E*-values and predicted length using the tool cath-resolve-hits (https://cath-tools.readthedocs.io/en/latest/tools/cath-resolve-hits/).

### IgSF domain searches with new HMM profiles

For comprehensive identification of IgSF domains in the *B*. *glabrata* genome, we employed a Hidden Markov Models (HMM) search using customized probabilistic models or HMMs profiles built with IgSF domain sequences identified from an InterProScan search [66,69]. Based on the initial predicted IgSF domain sequences extracted from InterProScan output, IgSF domains were grouped into clusters with less than 30% identity as a cutoff value. For clusters of IgSF domains containing more than two sequences, multiple sequence alignments using Clustal Omega v1.2.4 [70] were performed. Additionally, the profile HMMs using the hmmbuild command in HMMER 3.2.1 [71] was followed. The HMMs profiles were visualized to generate logos using Skylign [72]. Only those HMMs consensus profiles containing an intrachain cysteine loop of 40 aa or greater in size were selected for search against *B*. *glabrata* protein sequences using the hmmsearch command in HMMER package [73]. The 40 aa cut-off was chosen because to date all records of Ig domains in Pfam database had a minimum of 41 aa as the size of the cysteine loop. IgSF HMM profiles which hit with conditional *E*-values (c*E*-value) less than 0.001 were selected as candidate signatures for IgSF domains. IgSF domains were assigned only if a predicted IgSF signature contained a complete cysteine loop.

### Identifying additional FREPs based on genomic context and transcriptome data

The current genome assembly contained some inconsistencies that required consideration of BAC-derived genomic sequence data [38,74]. Furthermore, the genome-wide prediction analysis extended to upstream and/or downstream sequences, yielding more complete sequences for 14 of the previously known FREPs.

Considering the possibility of errors in annotation in the current assembly of the *B*. *glabrata* genome, we also manually checked sequences that were identified by the InterProScan and the new IgSF HMM profiles searches against available genomic sequences of FREP genes. This avoided potential errors in automated genome annotation that might call one gene as two separate genes. For each predicted partial gene/protein in this study, the flanking gene’s domain architecture was manually checked for presence of domains that could contribute to a complete FREP structure. Additionally, assembled transcripts from available RNA-Seq studies helped to capture longer or complete versions of particular FREPs, among them transcripts obtained from 12 different *B*. *glabrata* organs [32]. The coding sequence of each predicted FReD gene in this study was blasted against the assembled transcripts from the BB02 genome [32] using BLASTn with cut-off value ≥95% identity and aligned length ≥150 nt.

### FREP class assignment based on similarity to published FREPs

Protein sequences for FREP candidates were searched using BLASTp against all available FREPs proteins sequences in *B*. *glabrata* [30,32–34]. Candidate FREPs were assigned to a particular class using a cut-off value for BLASTp search of ≥90% (e.g. FREP12) or assigned as “class-like” with the cutoff value 85~90% (e.g. FREP4-like).

### Genomic location of FReDs in *B*. *glabrata*

Scaffold coordinates of FReDs identified in this study were downloaded from VectorBase [75] using the integrated BioMart web tool with *B*. *glabrata* VectorBase ID. The genomic distributions of FReDs were illustrated using the R package ggplot2 [76] with genomic location specified for IgSF and FBG domains.

### Relationship analysis, domain architectures and gene structures of FReDs

Full-length protein sequences of all identified FReDs were used to construct a maximum likelihood tree through IQ-TREE [77] with standard model selection followed by tree inference of 1000 bootstrap replicates. Conservative FBG domain predictions from InterProScan and of IgSF domains from new HMM profiles were merged using the cath-resolve-hits tool [78]. Domain architectures and gene structures of FReDs were drawn along the FReD gene tree using R packages ggplot2 v3.2.1 [76], ggtree v1.14.6 [79] and ggpubr v0.2.2 [80].

### Search for FReDs with binding affinity to early developing larval *S*. *mansoni* proteins within susceptible and resistant *B*. *glabrata* snails

Wu et. al (2017) identified peptides from plasma of SUS (NMRI) and RES (BS-90) *B*. *glabrata* strains eluted from affinity columns enriched with *S*. *mansoni* sporocyst membrane- and larval transformation proteins [49]. All data for sporocyst-bound snail peptides from Wu et al. [49], were blasted against predicted FReD proteins from this study. Matches of full peptide sequences with 100% identity from either strain of *B*. *glabrata* were identified and highlighted on our gene structures of the FReD proteins.

### Pairwise BLAST identity analysis of IgSF1, IgSF2, or FBG domains in *B*. *glabrata*

To compare sequence diversities within IgSF1, IgSF2, or FBG domains from *B*. *glabrata* FREPs, we applied pairwise blast comparisons. Only complete FReD genes with an ATG start codon (Methionine, M), a complete FBG domain and stop codon were included in this analysis. Separate pairwise blast identity analysis of all IgSF1, IgSF2 or FBG domain protein sequences from 2-IgSF FREPs were calculated based on all-against-all BLASTp top hits. The symmetric distance of tree topology was measured [81] and calculated and visualized with webtool Phylo.io [82]. Violin plots within R [83] were generated to compare sequence diversity of FBG and IgSF domains.

### Analysis of association of IgSF and FBG domains in *B*. *glabrata*

To investigate the association of IgSF domains with one another in 2-IgSF FREPs, or the associations of IgSF domains with FBG domains in FREPs, maximum likelihood trees were built using IQ-TREE with available protein sequences for IgSF1, IgSF2, and FBG domains. First, all IgSF domain sequences generated with new HMM profiles were used to build a tree to evaluate the ancestral associations among all IgSF domains in *B*. *glabrata*. The two IgSF domains from 2-IgSF FREP genes (IgSF1 and IgSF2) were highlighted in the tree. Secondly, protein sequences of FBG, IgSF1 and IgSF2 domains of 2-IgSF FREPs were used separately to build a tree of IgSF1 vs. IgSF2, matched tree of IgSF1 vs. FBG, and IgSF2 vs. FBG.

### Snail infection experiments and RNA-Sequencing study

To investigate the expression levels of FReDs in *B*. *glabrata* snails with or without *S*. *mansoni* exposure, a comprehensive transcriptomics analysis was carried out. Snails and experimental treatments used in this study are described in Lu et al. [84]. Briefly, uninfected juveniles (5-8mm in diameter) of lab-reared RES (BS-90) and SUS (M line) *B*. *glabrata* were exposed individually to 20 PR1 strain *S*. *mansoni* miracidia per snail. Control snails were treated similarly but were not exposed to *S*. *mansoni* miracidia. Snails of each group were reared in aerated aquaria containing at 25–27 °C and fed with lettuce [18]. At 0.5-, 2-, 8- and 40-days post-exposure (dpe), snails were collected and individually preserved in TRIzol reagent (Invitrogen) and stored at -80 °C until extraction. Biological replicates (3~6/group) were paired-end sequenced (2x 150 base reads) for each group using an Illumina NextSeq 500 instrument (Illumina, Carlsbad CA) at the Molecular Biology Facility, Biology Department, the University of New Mexico. Each replicate represents one snail sample. There were 56 snails used in this RNA-Seq study. An overview of snail samples per group and sequencing information is summarized in S1 Table. See Lu et al. [84] for a detailed description of sample collection, RNA and DNA extraction, library preparation, and differential gene expression analyses. Genes were considered differentially expressed in comparisons with cutoff values using fold change (FC) ≥2 (absolute log2FC ≥1), and a posterior probability of differential expression (PPDE) ≥0.95 for EBSeq analysis. Although some genes are differentially expressed in comparisons (absolute log2FC ≥1), they may not have been considered DE due to variations of replicates with PPDE <0.95. The raw RNA sequencing data are available at NCBI under SRA accession: PRJNA591872.

### Analysis of variant transcriptome sequences for some *B*. *glabrata* FREPs

Transcriptome variations including single nucleotide variants (SNV) were called using bcftools 1.9 (http://samtools.github.io/bcftools/) based on the pooled high quality RNA-Sequencing reads from both SUS and RES *B*. *glabrata*, followed by mapping to the *B*. *glabrata* BB02 reference genome [32]. The SNV analysis focused on coding regions of FREP genes of particular interest and used cut-off scores of quantity >10 and quality scores of filtering variants of >20. All variations were annotated and classified into categories including synonymous, nonsynonymous, splice region variants (a genetic alteration in the DNA sequence that occurs at the boundary of an exon and an intron, splice site), and stop gained variants by using Ensembl Variant Effect Predictor [85]. For SNVs within exons, homologous coding sequences for SUS and RES strains were determined mainly based on synonymous and nonsynonymous variants, and then translated to protein sequences. For each FREP gene of interest, protein sequences from the reference BB02 genome, and inferred homologous SUS and RES strain sequences were aligned together using the multiple sequence alignment program MAFFT [86] and visualized using JalView 2.11.0 [87]. Additionally, sequences of peptides of BgFREP2 and BgFREP3.3 from the plasma of SUS and RES strains obtained by liquid chromatograph-tandem mass spectrometry (LC-MS/MS) following different pull-down experiments [47], and sequences of peptides from the protomics study by Wu et al. (2017) [49] were compared to the deduced protein sequences we obtained.

### Other analyses

Table summary and figure generation were performed within R [88] and Bioconductor [89], including the following packages: ggplot2 [76], reshape2 [90], and openxlsx v4.1.0.1 [91]. Intermediate data analysis was done with an in-house parallel pipeline facilitated with GNU Parallel tool [92].

## Results

The definitions and abbreviations provided in Fig 1 apply below. We abbreviate the Vectorbase ID of genes to the extent possible for the sake of readability: for instance, “BGLB000152” is referred to as “Bg152”, “BGLB000011” is referred to as “Bg11”, and so on. With respect to the alternative splicing in *B*. *glabrata* FReDs, multiple proteins encoded by the same gene were differentiated by protein IDs, with “-PA”, “-PB”, “-PC”, “-PD” or “-PE” suffix following the gene ID. In this study, some gene/protein sequences extended by manual checking are referred to as, for instance, “Bg152*”/ “Bg152-PA*” to differentiate them from the original version of “Bg152”/ “Bg152-PA” in Vectorbase.

The criteria for “complete” FREP and sFReD were set differently here because whereas little is known about sFReDs and their complete structures, FREPs are known to be secreted proteins and require a signal peptide. We defined a “**complete FREP**” as having a signal peptide, start codon, stop codon, a complete FBG domain, and 1 or 2 IgSF domain(s) each with cysteine loop >40 aa. If any of the above components could not be verified, a FREP was considered to be a “partial” FREP. We defined a “**complete sFReD**” as having a start codon, stop codon and a complete FBG domain. The FBG domain begins with a **SCRDV** like motif, contains prosite motif **PS00514 W-W-[LIVMFYW]-x(2)-C-x(2)-[GSA]-x(2)-N**-G, and a **EMKIRE** like motif just preceding a stop codon, according to the FBG domains initially described in FREPs in *B*. *glabrata* [20]. Similary, if any of these components could not be verified, a particular sFReD was considered to be a “partial” sFReD.

### Prediction of FBG and IgSF domains in *B*. *glabrata*

**Fig 2:**
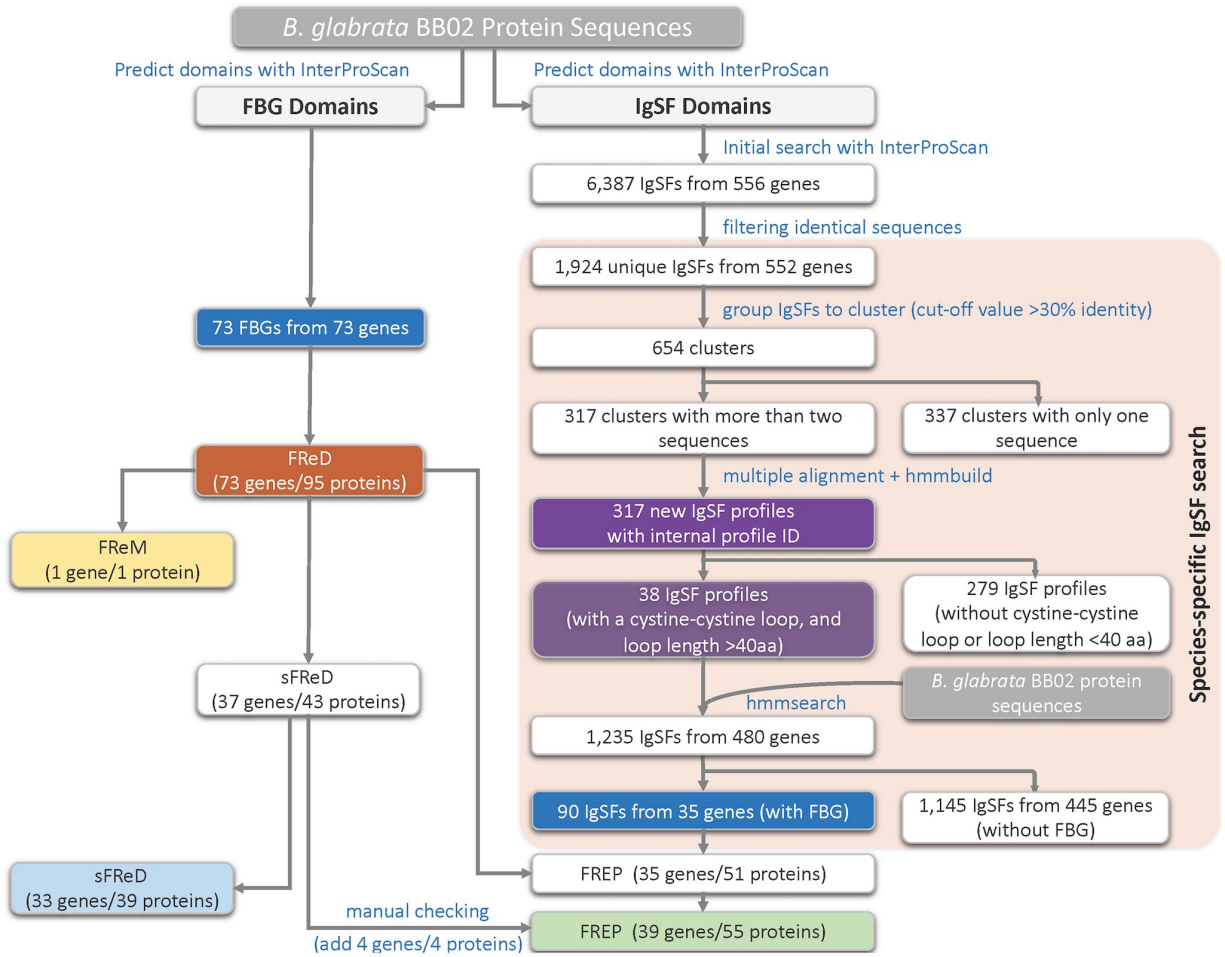
Workflow to predict FBG and IgSF domains in the genome assembly of BB02 *B*. *glabrata*. The colored boxes highlight some of the important steps/results in the workflow.

InterProScan was used to detect both FBG and IgSF domains in the genome assembly (Fig 2). As shown on the left side of the figure, a total of 73 genes with an FBG domain were found, estimated to encode 95 different proteins (through alternative splicing). Among the 73 genes, 34 did not contain an IgSF domain, of which, 33 genes were designated as sFReDs, and one as a FReM. The remaining 39 genes, including four confirmed via manual checking (S2 Table), were associated with an IgSF domain so fit the definition of being FREPs. The pipeline for identification of IgSF domains is shown on right side of Fig 2. We identified 35 FREP genes encoding 51 proteins (through alternative splicing) with this approach, again harmonized to 39 based on manual checking (green bar at bottom of Fig 2). The 39 FREPs are summarized in Table 1 including extent of completeness, number of IgSF domains, and published or newly identified in this study (S1 Fig).

**Table 1:** Summary of 39 FREPs discussed in this study.

	Previously reported FREPs		Newly identified FREPs		Total
	1-IgSF	2-IgSFs	1-IgSF	2-IgSFs	
Complete	6*	19*	1	0	**26**
Partial	1	6	4	2	**13**
Total	**7**	**25**	**5**	**2**	**39**
	**32**		**7**		

*: Some previously published FREPs were extended in this study as complete FREPs.

### Summary of predicted FReDs in *B*. *glabrata* genome

Additional details for the 95 proteins predicted for the 73 FReD genes (through alternative splicing) identified are provided in Table 2 and S2 Table. Genes with absence of an ATG start codon or stop codon were designated as “partial.” One partial FReM gene (Bg40437) is confirmed, which is composed of a N-terminal region, a middle epidermal growth factor (EGF)-like region, and a partial C-terminal FBG domain (missing C-terminal EMKIRE like motif of FBG,) [43].

**Table 2:** Summary of predicted FReD proteins in *B*. *glabrata* BB02 strain.

ID in text	NCBI annotation	Type of FReD	Best hit to published FREP
Bg11-PA	Fibrinogen-like protein A, partial	partial 1-IgSF FREP	11_sig_1434_BGLB000011
IBg11-PB	Fibrinogen-like protein A, partial	1-IgSF FREP	11_sig_1434_BGLB000011
Bg19-PA	Fibrinogen C domain-containing protein 1-B-like	2-IgSF FREP	FREP5
Bg19-PB	Fibrinogen C domain-containing protein 1-B-like	2-IgSF FREP	FREP5
Bg21-PA	Fibroleukin-like	partial 2-IgSF FREP	17_tig_2702_BGLB000021
Bg21-PB	Fibroleukin-like	2-IgSF FREP	17_tig_2702_BGLB000021
Bg74-PA	Uncharacterized protein LOC106071720, partial	partial 2-IgSF FREP	FREPJ4
Bg74-PB	Uncharacterized protein LOC106071720, partial	partial 2-IgSF FREP	FREPJ4
Bg96-PA	Fibroleukin-like	2-IgSF FREP	FREP3.1
Bg96-PB	Fibroleukin-like	2-IgSF FREP	FREP3.1
Bg100-PA	Angiopoietin-4-like	2-IgSF FREP	14_tig_2094_BGLB000100
Bg100-PB	Angiopoietin-4-like	2-IgSF FREP	14_tig_2094_BGLB000100
Bg116-PA	Uncharacterized protein LOC106057350	2-IgSF FREP	1_tig_99_BGLB000116
Bg116-PB	Uncharacterized protein LOC106057350	2-IgSF FREP	1_tig_99_BGLB000116
Bg131-PA	---	partial 2-IgSF FREP	z_tig_6870_frameshifts
Bg133-PA	Angiopoietin-2-like	2-IgSF FREP	22_tig_4104_BGLB000133
Bg133-PB	Angiopoietin-2-like	2-IgSF FREP	22_tig_4104_BGLB000133
Bg140-PA	Fibroleukin-like	2-IgSF FREP	FREP13.1
Bg140-PB	Fibroleukin-like	2-IgSF FREP	FREP13.1
Bg141-PA	Fibrinogen-like protein A	1-IgSF FREP	FREPA
Bg141-PB	Fibrinogen-like protein A	1-IgSF FREP	FREPA
Bg152-PA	Fibrinogen C domain-containing protein 1-like	1-IgSF FREP	FREP4
Bg152-PB	Fibrinogen C domain-containing protein 1-like	1-IgSF FREP	FREP4
Bg179-PA	Fibrinogen beta chain-like	1-IgSF FREP	FREP1
Bg179-PB	Fibrinogen beta chain-like	partial 1-IgSF FREP	FREP1
Bg204-PB	Tenascin-R-like	2-IgSF FREP	FREP3.2
Bg4529-PB	Uncharacterized protein LOC106059401	2-IgSF FREP	FREP7-like
Bg4530-PB	Fibrinogen C domain-containing protein 1-B-like	2-IgSF FREP	FREPJ2-like
Bg5224-PA	Uncharacterized protein LOC106061038	partial 2-IgSF FREP	FREPJ5
Bg5224-PB	Uncharacterized protein LOC106061038	2-IgSF FREP	FREPJ5
Bg6034-PB	Uncharacterized protein LOC106062908	partial 2-IgSF FREP	FREP12.1-as2-like
Bg7076-PB	Angiopoietin-related protein 2-like	2-IgSF FREP	4_tig_565_BGLB000073-like
Bg7576-PB	Uncharacterized protein LOC106066900 isoform X2	2-IgSF FREP	FREPM
Bg7576-PC	Uncharacterized protein LOC106066900 isoform X1	2-IgSF FREP	FREPM
Bg10948-PB	Uncharacterized protein LOC106074953	partial 2-IgSF FREP	FREPK1
Bg11626-PA	Angiopoietin-related protein 2-like	partial 2-IgSF FREP	FREP12-like
Bg11626-PB	Angiopoietin-related protein 2-like	2-IgSF FREP	FREP12-like
Bg11627-PA	Angiopoietin-related protein 2-like	partial 1-IgSF FREP	5_tig_565_BGLB011626
Bg11627-PB	Angiopoietin-related protein 2-like	2-IgSF FREP	5_tig_565_BGLB011626
Bg14428-PB	Ryncolin-4-like	1-IgSF FREP	FREP14
Bg16934-PA	Fibrinogen beta chain-like	2-IgSF FREP	20_tig_3569_BGLB009245b
Bg16935-PA	Angiopoietin-4-like	2-IgSF FREP	19_tig_3569_BGLB009245a
Bg17688-PA	Fibrinogen beta chain-like	2-IgSF FREP	zb_tig_15796
Bg17893-PA	Uncharacterized protein LOC106053933	2-IgSF FREP	6_tig_990_BGLB014388
Bg20516-PA*	Fibrinogen C domain-containing protein 1-like	partial 2-IgSF FREP	new partial FREP
Bg22646-PA	Fibrinogen C domain-containing protein 1-B-like, partial	partial 1-IgSF FREP	new partial FREP
Bg23857-PA	Fibrinogen C domain-containing protein 1-like, partial	partial 1-IgSF FREP	new partial FREP
Bg25144-PA*	Ficolin-1-like	partial 2-IgSF FREP	new partial FREP
Bg25334-PA	Fibrinogen C domain-containing protein 1-like	partial 1-IgSF FREP	FREPJ5
Bg28617-PA	Fibrinogen-like protein A	1-IgSF FREP	FREP2
Bg29167-PA	Tenascin-like	partial 1-IgSF FREP	new partial FREP
Bg32905-PA*	Ficolin-2-like	partial 2-IgSF FREP	FREPJ1
Bg38205-PA	Angiopoietin-4-like	partial 1-IgSF FREP	new partial FREP
Bg40226-PA*	Techylectin-like protein	Partial 2-IgSF FREP	FREPJ3-like
Bg40228-PA	Fibroleukin-like	1-IgSF FREP	new FREP
Bg177-PA	Microfibril-associated glycoprotein 4-like	sFReD	
Bg177-PB	Microfibril-associated glycoprotein 4-like	sFReD	
Bg178-PA	Fibrinogen C domain-containing protein 1-like	sFReD	
Bg178-PB	Fibrinogen C domain-containing protein 1-like	sFReD	
Bg2450-PB	Ryncolin-1-like	sFReD	
Bg2494-PB	Ficolin-2-like isoform X1	sFReD	
Bg2494-PC	Ficolin-1-like isoform X3	sFReD	
Bg2494-PD	Ficolin-2-like isoform X2	sFReD	
Bg2494-PE	Ficolin-1-like isoform X4	partial sFReD	
Bg5196-PB	Angiopoietin-related protein 1-like	partial sFReD	
Bg12039-PB	Ficolin-1-like, partial	partial sFReD	
Bg12226-PB	Techylectin-5B-like	partial sFReD	
Bg12382-PB	Angiopoietin-4-like	partial sFReD	
Bg14428-PA	Ryncolin-4-like precursor	partial sFReD	FREP14
Bg16307-PA	Ficolin-2-like	sFReD	
Bg16436-PA	Angiopoietin-related protein 6-like	partial sFReD	
Bg17041-PA	Fibrinogen C domain-containing protein 1-like	sFReD	
Bg17605-PA	Ficolin-2-like	sFReD	
Bg18336-PA	Fibrinogen-like protein A	sFReD	za_tig_4714
Bg20351-PA	Ficolin-2-like	sFReD	BgMFREP4_AY012701-like
Bg21912-PA	Ficolin-2-like	sFReD	
Bg22867-PA	Fibrinogen beta chain-like, partial	partial sFReD	
Bg25627-PA	Ficolin-1-like	partial sFReD	
Bg25643-PA	Microfibril-associated glycoprotein 4-like	sFReD	
Bg28385-PA	Ficolin-2-like	sFReD	
Bg29288-PA	Uncharacterized protein LOC106051237	partial sFReD	
Bg30478-PA	Fibroleukin-like, partial	partial sFReD	
Bg30830-PA	Ryncolin-1-like, partial	partial sFReD	
Bg31381-PA	Ficolin-1-like	sFReD	
Bg31596-PA	Angiopoietin-related protein 6-like, partial	partial sFReD	
Bg34525-PA	Techylectin-5A-like	partial sFReD	
Bg36251-PA	Ficolin-2-like	sFReD	
Bg37621-PA	Angiopoietin-1-like	partial sFReD	
Bg38117-PA	Fibrinogen C domain-containing protein 1-like, partial	partial sFReD	
Bg38186-PA	Ficolin-1-like	sFReD	
Bg38723-PA	Ficolin-1-like	sFReD	
Bg39918-PA	Ficolin-2-like	sFReD	
Bg40034-PA	Ficolin-1-like, partial	partial sFReD	16_tig_2402_BGLB007591-like
Bg40339-PB	Ficolin-2-like	sFReD	
Bg40437-PA	Uncharacterized protein LOC106061221	partial FReM	FReM

For readability, the dashed lines in Table 2 separate the three types of FReDs: FREP, sFReD, or FReM.

**Notes**:

In the column of “**ID in text**”, multiple proteins encoded by the same gene due to alternative splicing were differentiated by different version of protein, such as “-PA”, “-PB”, “-PC”, “-PD” or “-PE” suffix following the gene ID;

In the column of “**NCBI Annotation**”, “**---**” represents no available annotation for a particular protein;

In the column of “**Type of FReD”**, each particular FReD was considered as complete or partial based on the criteria set in this study above; “1-IgSF FREP” and “2-IgSF FREP” represent the number of IgSF domains present in a particular FREP;

“**Best hit to published FREP**” represent the best hit of individual protein against published *B*. *glabrata* FREP sequences from available sources [30,32–34]: best hit of protein sequence identity ≥90% is considered as the same FREP; identity 85~90% is considered as “-like” of the corresponding FREP; identity <85% is not assigned to any known FREP.

In the “**Best hit to published FREP**” column, several FREPs were emphasized in this study were highlighted in yellow.

Of the 39 FREP genes (Fig 2, Table 1), 26 were considered as complete and 13 were partial. In addition to FREPs noted in previous studies [30,32–34], two new FREPs were found. The first one is a 1-IgSF FREP (Bg40228) and has an ATG start codon, a signal peptide of 19 aa, a complete IgSF1 (114 aa), a truncated IgSF2 (only 26 aa), an interceding region, and a full length FBG (203 aa) domain, which is considered as a complete “new FREP”. The second is a partial 2-IgSF FREP (Bg20516) that lacks an ATG start codon, but is otherwise complete with a 23 aa signal peptide, and complete domains of IgSF1 (114 aa), IgSF2 (120 aa), an ICR, and FBG (202 aa), which is considered as a “new partial FREP”. Five more genes with both FBG and IgSF domains were found for the first time, but no signal peptides could be identified for these genes in the current assembly. These five genes are designated as “new partial FREP” in Table 2 and await future study.

In addition, we also extended or clarified 18 previously known FREP sequences. For instance, 26aa including a signal peptide were added to the N-terminus of Bg179. The protein sequences for some previously reported FREPs were extended to include N- or C-termini. (Table 1). These improvements provided signal peptides for 10 published FREPs and FBG or IgSF2 domain sequences for 5 published FREPs (see more details in S3 Table). We note that FREP3.3 [47] is not on our list because its key portions were found on different scaffolds, precluding full annotation. The sequence and structure of this gene may be completed with future improved genome assembly.

### Summary of domain architecture of FReDs in *B*. *glabrata*

**Fig 3:**
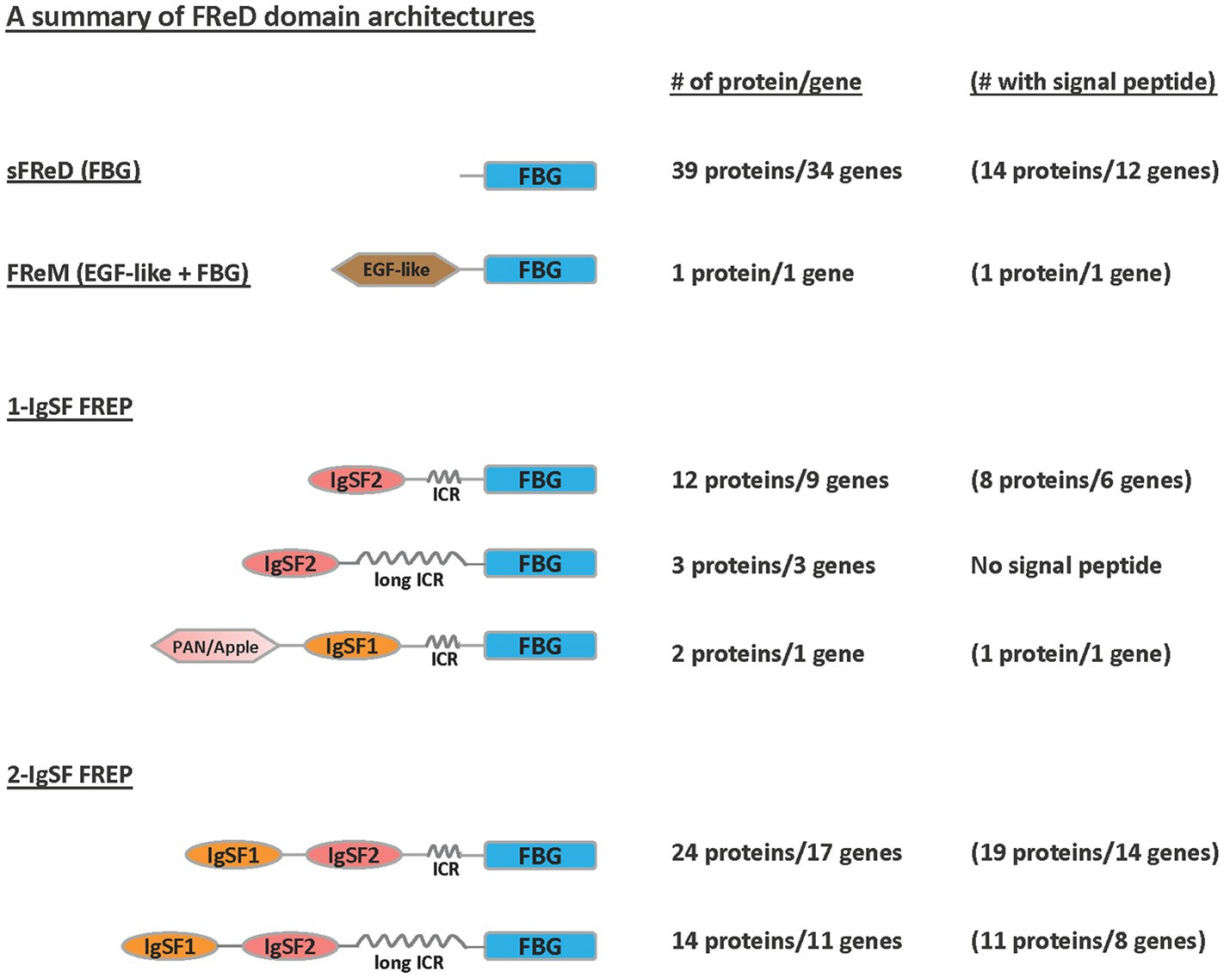
Predicted domain architectures of FReD proteins in *B*. *glabrata* BB02. Three main categories are indicated: **sFReD (FBG), FReM (EGF-like + FBG), 1-IgSF FREP (1 IgSF loop + FBG)**, and **2-IgSF FREP (2 IgSF loops + FBG). FBG**: fibrinogen domain; **EGF-like**: epidermal growth factor (EGF)-like domain; **PAN/Apple**: PAN/Apple domain; **IgSF1**: Immunoglobulin Superfamily Member 1; **IgSF2**: Immunoglobulin Superfamily Member 2; **ICR**: interceding region (<150aa) between IgSF and FBG domains that form coiled coils; **long ICR**: long interceding region (150~320aa).

Some FReD genes may encode proteins with more than one type of domain architecture (Fig 3). The PAN/Apple domain in a 1-IgSF FREP gene (Bg179) previously identified in *B*. *glabrata* genome [32] was confirmed here. This FREP gene is unusual for containing an IgSF1 domain next to the ICR rather than the IgSF2 domain typically found in this location. It is noticed that long sequences of coiled coils (150~320aa) were detected between IgSF and FBG domains in some FREPs and are considered as long interceding regions (long ICR) in the present study.

### Analysis of relationships among *B*. *glabrata* FReD genes

**Fig 4:**
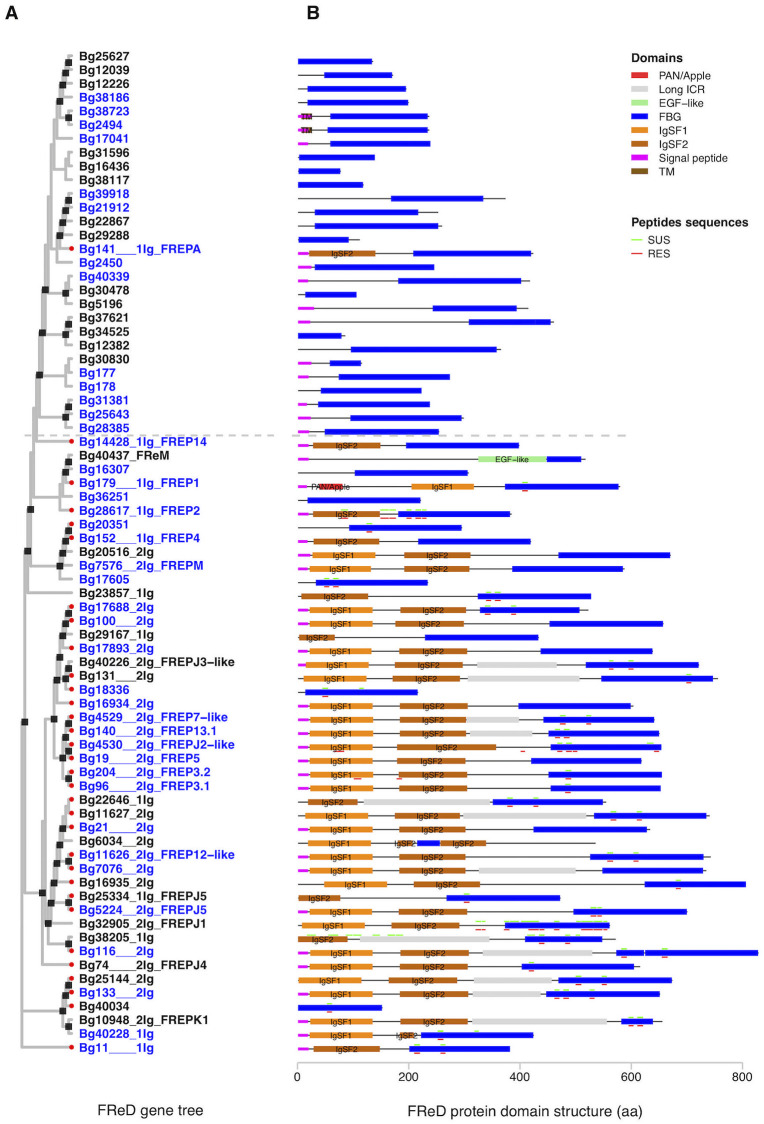
Gene tree and representative protein domain architecture of 73 *B*. *glabrata* FReDs. **A**. Maximum likelihood tree with 1000 bootstrap tests was constructed using full length representative protein sequences of 73 FReD genes in *B*. *glabrata*. Nodes with bootstrap support of 75 or higher are marked with black squares. The gene IDs of the 73 FReDs were followed with number of IgSF domains and the best hit with any published FREP genes (BLASTp hit with at least 150 aa aligned with ≥ 90% identity was considered as the same FREP; or considered a “FREP-like” gene if identity was 85% ~ 90%; identity <85% was not assigned to any published FREP). Any FReD genes with best hit to FREPs from the genome paper [32] are labeled with a red dot at the corresponding gene ID. Complete FREP or FReD genes fitting the criteria in this study were highlighted in blue text. **B**. Domain architectures were predicted using InterProScan and lineage specific HMM models. “**Peptide sequences**” represent multiple peptide sequences extracted from a proteomics study [49]. Colored small horizontal bars above or below some protein domain structures highlight the matched location of peptide sequences with binding affinity to *S*. *mansoni* sporocyst membrane-enriched and larval transformation proteins, from either schistosome-susceptible NMRI strain (SUS in green) or -resistant BS-90 strain (RES in red) of *B*. *glabrata*. The dashed gray line was manually added to distinguish most “sFReD clades” from “FREP clades”.

To compare the 73 FReD genes predicted in *B*. *glabrata*, a gene tree with full length sequences of the FReDs was constructed (Fig 4A). As the sequences being compared vary considerably in length, the general purpose of this tree was to show a broad overview of relationships among all FReDs (alternative version of the tree showing only FBG domains is provided in S2 Fig). For each gene, the predicted domain structure (Fig 4B) and gene exon intron structure (S4C Fig) of representative protein is also provided. Two 1-IgSF FREPs, Bg28617 (FREP2) and Bg14428 (FREP14), were both found to have only one exon (S4C Fig). Although poly-A tails were not found within the exon for either FREP, the presence of a single exon coding for both IgSF2 and FBG domain suggests a history of retrotransposition for both.

As expected because of the manner of tree construction, most sFReDs grouped together (see above the dashed line which is included as a convenient reference point) as did most FREPs (below the line). We noted that 11 sFReD genes above the dashed line were located near (less than 5kb) an edge of a scaffold within the genome assembly (S3 Fig). Although some of them have a predicted signal peptide or ATG start codon, we cannot rule out the possibility that some sFReDs are actually part of complete FREP genes, which contain upstream IgSF domains located on another scaffold. One such example is sFReD gene Bg40034 which was located on a 1.12kb scaffold (S3 Fig).

**Fig 5:**
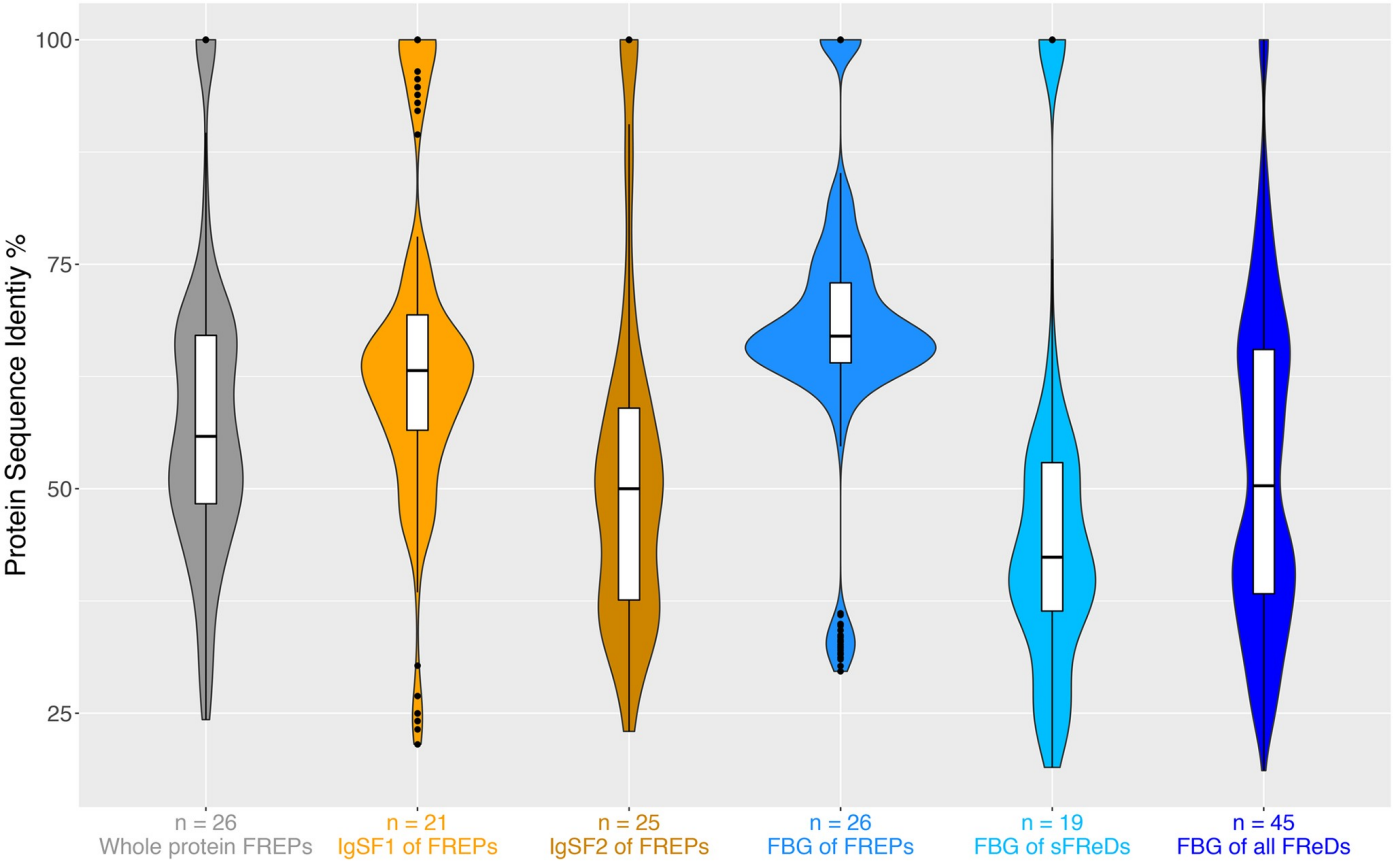
Violin plots of protein sequence-pairwise identity analyses of IgSF1, IgSF2, and FBG domains in *B*. *glabrata* FReD genes. The 26 complete FREP protein sequences, and complete FBG domain sequences from all FReD genes in *B*. *glabrata* were used for pairwise blast identity analysis. Number under each Violin plot at x-axis is the number used for each assigned analysis, and y-axis represents percentage of protein sequence identity (%).

A single 1-IgSF FREP (Bg141) falls above the dashed line nesting among sFReDs. This FREP is divergent from others in at least two ways. Its IgSF2 domain has only about 30% protein sequences identity to the IgSF2 domains in other FREPs, placing it in the lowest quartile (*Q1* = 37.6%) of all IgSF2 pairwise comparisons in FREPs (Fig 5C, S4 Table). Similarly, its FBG domain had ~60% identity protein sequences to FBG domains in all other FREPs, also placing it in the lowest quartile (*Q1* = 64%) of all FBG pairwise comparisons in FREPs (Fig 5D, S4 Table). Also, 6 sFReD genes and the FReM gene (Bg40437) fall among the FREPs (below the dashed line) and as they are not near scaffold edges, would appear not to be parts of FREPs yet to be fully resolved. Their scattered locations on the tree suggest separate origins or derivations from the FBG domains of neighboring FREPs.

Highlighting some of the better-known FREPs, there is some tendency for those 1-IgSF FREPs to cluster together (note especially FREP1, 2 and 4). Similarly, FREP3.1, 3.2, 5, 7 and 13 (2-IgSF FREPs) formed a cluster on the tree. Noteworthy is that the remaining FREPs were scattered amongst several clusters on this tree suggesting separate orgins and functionalities.

### Peptide sequences match analysis

Among the predicted protein domains in Fig 4B, 40 proteins encoded by 30 FReD genes have absolute matches to peptide sequences derived from whole plasma proteins of SUS (NMRI) or RES snails found to bind to early developing *S*. *mansoni* sporocyst tegmental membrane proteins or larval transformation proteins [49]. Here it should be noted that the peptides retrieved do not necessarily comprise the exact region of the proteins binding to the sporocysts, but are the ones successfully retrieved following their sampling protocol [49]. These peptides matched several of our predicted FBG, IgSF1, IgSF2, regular ICR, or long ICR regions, particularly so for FBG domains. Several peptides along much of the length of Bg38205 were retrieved only from SUS snails whereas in RES snails, two peptides found in the IgSF1 and IgSF2 regions of FREP3.2 were distinctive (Fig 4).

### Summary of pairwise BLAST identity analyses of IgSF1, IgSF2, and FBG domains in *B*. *glabrata*

**Fig 6:**
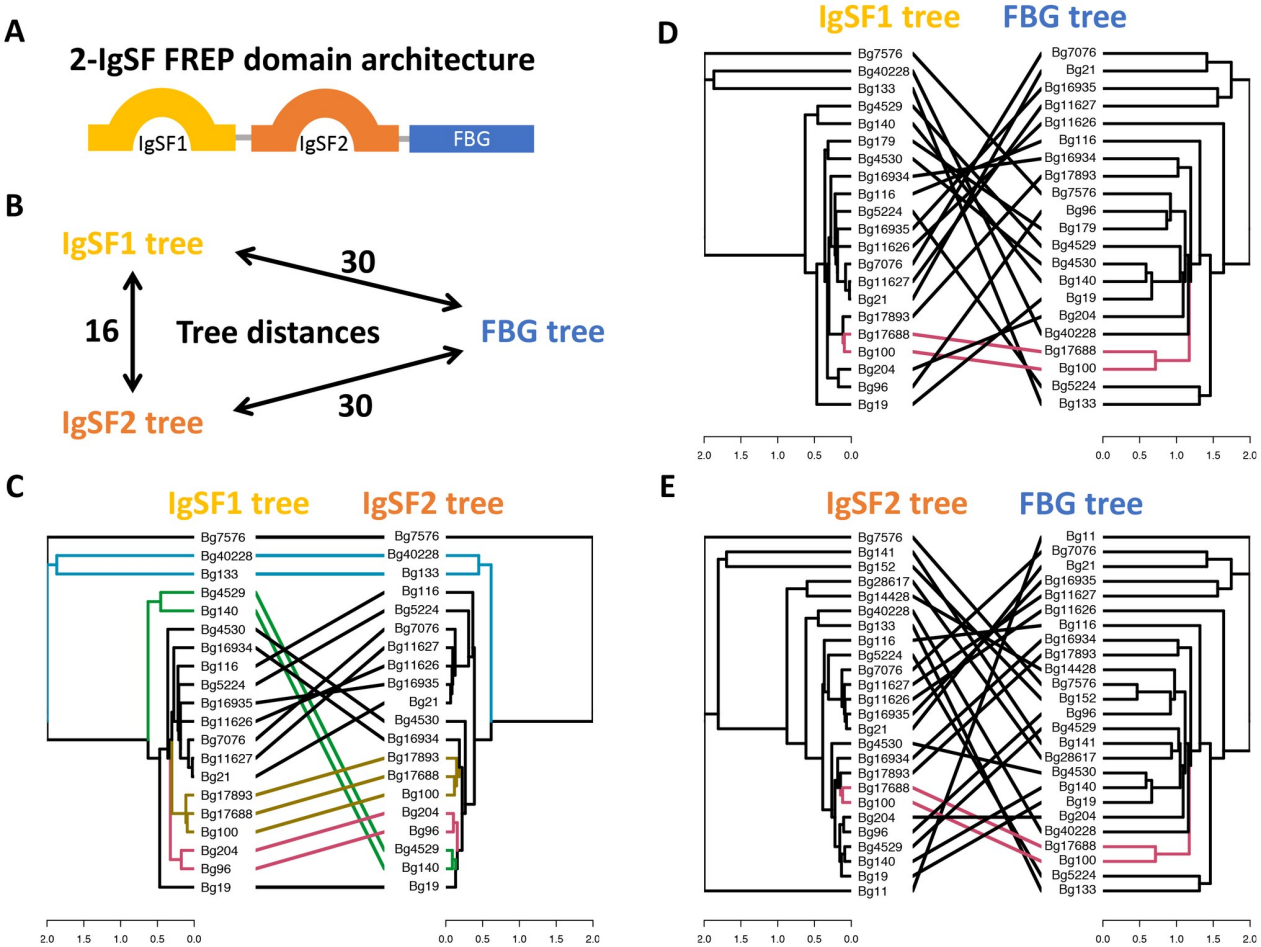
Domain tree comparisons of IgSF1, IgSF2 and FBG domains from complete 2-IgSF FREPs in *B*. *glabrata*. A. Domain structure of 2-IgSF FREPs; **B**. Tree distances were measured by Robinson-Foulds or symmetric distance among trees of IgSF1, IgSF2 and FBG domains from 2-IgSF FREPs; **C**. Tanglegram plot of a side by side ML (Maximum Likelihood) trees for IgSF1 and IgSF2 domains from 2-IgSF FREPs; **D**. Tanglegram plot of ML trees for IgSF1 and FBG domains from the 2-IgSF FREPs; **E**. Tanglegram plot of ML trees for IgSF2 and FBG domains from the 2-IgSF FREPs. The colored connection lines in 6C, 6D and 6E highlighted some common subtrees.

To trace the associations of IgSF1, IgSF2 and FBG domains, 19 available complete 2-IgSF FREPs (Table 1) with all three domains were selected (Fig 6A and 6B).

The symmetric distance between the IgSF1 tree and the IgSF2 tree (16) is closer than either the distance between IgSF1 tree and FBG tree (30) or the distance between IgSF2 tree and FBG tree (30), measured by Robinson-Foulds distances. In Fig 6C, 6D, and 6E, separate trees were built based on the protein sequences of IgSF1, IgSF2, or FBG domains. Such trees for pairs of domains are shown side-by-side to highlight discordant relationships between two domains from the same FREP. More concordance is seen between IgSF1 vs. IgSF2 trees (Fig 6C) than between IgSF1 vs. FBG trees (Fig 6D) or IgSF2 vs. FBG trees (Fig 6E).

To compare sequence diversity of FBG, IgSF1 and IgSF2 domains in FReD genes, violin plots (Fig 5) were generated for complete FReD genes (S3 Table) based on the criteria in this study (Fig 1). Specifically, protein sequences for IgSF1, IgSF2 and FBG domains from the 26 complete FREPs, and complete FBG domain sequences from sFReD genes were analyzed with pairwise blast identity. Median values of FBG domain pairwise blast identity is 66.99 (S4 Table), significantly higher than for IgSF1 (63.16, non-parametric Mann–Whitney *U* test *p* = 2.02^−8^ or IgSF2 (50.0, *p* = 2.99^−41^), consistent with earlier observations [26,29,31]. FBG domains from FREPs were significant more conserved with respect to protein sequence identity than sFReDs (*p* = 9.41^−40^). See more details in S4 Table.

It is also noteworthy that there are 9 “outliers” with high pairwise identities in the violin plot of IgSF1 (Fig 5B). Some of the “outliers” are among a subgroup of 2-IgSF FREPs with long ICRs that clustered closely on the FReD gene tree (Fig 4A), including Bg21, Bg7076, Bg11626, Bg11627, and Bg16935.

### Subfamilies of IgSF domains in *B*. *glabrata*

**Fig 7:**
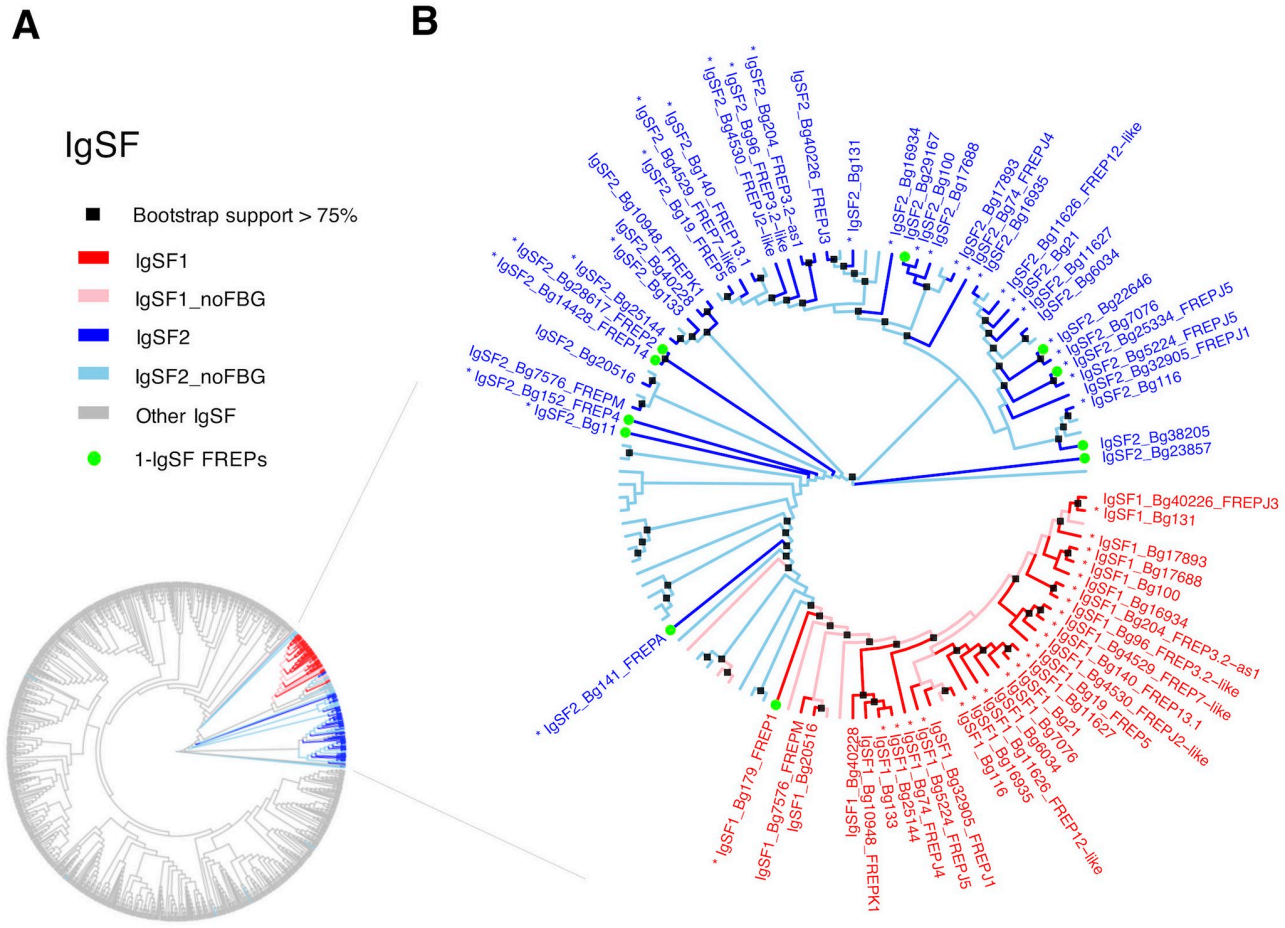
Unrooted Maximum Likelihood (ML) trees of IgSF domains identified in *B*. *glabrata* with new HMM profile search. **A**: ML tree with all IgSF domains from search using the 38 new IgSF HMM profiles (in Fig 2). Gray branches in panel A represent other IgSFs predicted with 36 new HMM profiles, which are not associated with FBG domain; **B**: An ML tree for only IgSF1 and IgSF2 domains from *B*. *glabrata*. ML trees with 1000 bootstrap tests were constructed using IQ-TREE. Nodes with bootstrap support of 75 or higher were highlighted with black squares at the nodes in panel B. Any domains associated with FBG domain are colored in red (IgSF1) or in blue (IgSF2) and followed with name of best hit to published FREPs. IgSF1 or IgSF2 domains without association with FBG domain are colored in pink (IgSF1) or in light blue (IgSF2). Gene has been identified previously in the genome paper [32] is labelled with an asterisk next to the corresponding gene ID. 1-IgSF FREPs are labeled with a green dot at the end of the branches. All FREPs identified in this study were named with IgSF domain, the Vectorbase ID and the best hit to published FREPs.

An unrooted Maximum Likelihood (ML) tree (Fig 7A) was generated based on all 38 IgSF new HMM profiles from the workflow in Fig 2. The two FREP-associated IgSFs (IgSF1 in red and IgSF2 in blue) clearly separated from the gray branches, which represent 1,145 IgSFs (445 genes) predicted from the 36 non-FREP-IgSFs HMM profiles (Fig 2).

IgSF1 and IgSF2 domains were used to generate a more detailed ML tree in Fig 7B. IgSF1 and IgSF2 domains were again clearly separable. Care is required in these interpretations because some of the IgSF2 domains not associated with FBG domains may reflect incomplete or erroneous annotation, or could be from other groups of molecules like CREPs for instance [33]. Notable is the fact that the majority of extant FREP genes (27 of the 39) have both IgSF1 and IgSF2 domains. With respect to IgSF1 domains, the peculiar FREP1, the only FREP to have a single IgSF domain that is of the IgSF1 category, falls on the outside of the IgSF1 group. Most of the remaining IgSF1 domains, all coming from 2-IgSF FREPs, cluster more closely together, suggestive of a separate diversification. With respect to IgSF2 domains, a cluster suggestive of a separate diversification is observed including all those FREPs such as FREPs 3, 5 and 7 known to be 2-IgSF FREPs. The IgSF2 domains associated with those 1-IgSF FREPs (FREPs 2 and 4 for instance) fall in adjacent, separate groups. As supported by Fig 6C, it suggests IgSF1 and 2 domains may have diversified in parallel in those 2-IgSF FREPs.

### Comparisons of expression levels of FReD genes in SUS (M line) or RES (BS-90) *B*. *glabrata*: Constitutive and post-exposure responses to *S*. *mansoni*

Transcriptomic profiles of *B*. *glabrata s*nails not exposed to *S*. *mansoni* were considered as representative of constitutive gene expression. For such snails, using SUS snails as a baseline, there are three time-matched comparisons with RES snails: 0.5-, 2- and 40 days (Table 3). The first two time points represent juvenile snails and the third represents sexually mature snails. The overall constitutive expression profiles for the different FReDs are mixed between the two snail strains. Four FReD genes (2 FREPs and 2 sFReDs) were constitutively higher in RES than SUS snails in at least 2 of the 3 comparisons, and 6 FReD genes (5 FREPs and 1 sFReDs) were constitutively higher in SUS than RES snails in at least 2 of the 3 comparisons.

**Table 3:** Summary of expression levels of FReD genes in RES and SUS *B*. *glabrata*: Constitutive and post-exposure responses to *S*. *mansoni*.

ID in text	Unexposed RES vs. Unexposed SUS			RES exposed to *S*. *mansoni*				SUS exposed to *S*. *mansoni*				Type of FReD	Best hit to known FREP
	0.5d	2d	40d	0.5d	2d	8d	40d	0.5d	2d	8d	40d		
Bg11	ns	ns	ns	1.31	ns	1	ns	ns	ns	ns	ns	1-IgSF FREP	11_sig_1434_BGLB000011
Bg19	ns	ns	-2.16	ns	ns	ns	ns	ns	ns	ns	ns	2-IgSF FREP	FREP5
Bg74	ns	1.51	ns	ns	ns	ns	ns	ns	ns	ns	ns	partial 2-IgSF FREP	FREPJ4
Bg96	ns	1.06	ns	ns	1.86	1.09	ns	ns	ns	ns	ns	2-IgSF FREP	FREP3.1
Bg116	ns	-2.11	ns	1.95	3.64	ns	ns	ns	ns	ns	ns	2-IgSF FREP	1_tig_99_BGLB000116
Bg131	ns	-1.74	-2.52	ns	ns	ns	ns	ns	ns	ns	ns	partial 2-IgSF FREP	z_tig_6870_frameshifts
Bg133	1.8	ns	ns	ns	ns	ns	ns	ns	ns	ns	ns	2-IgSF FREP	22_tig_4104_BGLB000133
Bg141	ns	ns	3.18	ns	ns	ns	ns	ns	ns	ns	ns	1-IgSF FREP	FREPA
Bg152	ns	ns	ns	2.93	9.18	4.91	3.37	-5.59	ns	ns	ns	1-IgSF FREP	FREP4
Bg204	-2.1	-2.58	-3.15	1.94	3.09	ns	ns	ns	ns	ns	ns	2-IgSF FREP	FREP3.2
Bg4529	ns	-1.06	ns	ns	1.07	ns	ns	ns	ns	ns	ns	2-IgSF FREP	FREP7-like
Bg4530	ns	-2.79	-3.88	ns	2.59	ns	ns	ns	ns	ns	ns	2-IgSF FREP	FREPJ2-like
Bg5224	ns	1.24	ns	1.13	1.45	ns	ns	ns	ns	ns	ns	2-IgSF FREP	FREPJ5
Bg6034	ns	-1.19	ns	ns	3.64	ns	ns	ns	ns	ns	ns	partial 2-IgSF FREP	FREP12.1-as2-like
Bg14428	ns	ns	-1.11	1.05	ns	ns	ns	ns	ns	ns	ns	1-IgSF FREP	FREP14
Bg16934	ns	ns	-5.41	ns	ns	ns	ns	ns	ns	ns	-6.42	2-IgSF FREP	20_tig_3569_BGLB009245b
Bg16935	1.04	ns	ns	ns	ns	ns	ns	ns	ns	ns	ns	2-IgSF FREP	19_tig_3569_BGLB009245a
Bg17688	ns	ns	-2.74	ns	ns	ns	ns	ns	ns	ns	ns	2-IgSF FREP	zb_tig_15796
Bg20516*	8.96	ns	ns	ns	6.79	4.84	ns	ns	ns	ns	ns	partial 2-IgSF FREP	new partial FREP
Bg22646	ns	ns	ns	ns	1.1	ns	ns	ns	ns	ns	ns	partial 1-IgSF FREP	new partial FREP
Bg23857	ns	-2.33	-2.53	1.4	2.42	ns	ns	ns	ns	ns	ns	partial 1-IgSF FREP	new partial FREP
Bg25144*	ns	ns	ns	ns	1.44	ns	ns	ns	ns	ns	ns	partial 2-IgSF FREP	new partial FREP
Bg25334	4.4	4.34	2.93	ns	1.4	ns	ns	1.78	ns	ns	ns	partial 1-IgSF FREP	FREPJ5
Bg28617	ns	ns	1.8	2.29	3.92	ns	ns	ns	ns	ns	2.48	1-IgSF FREP	FREP2
Bg29167	ns	ns	-2.3	ns	ns	ns	ns	ns	ns	ns	ns	partial 1-IgSF FREP	new partial FREP
Bg32905*	6.74	5.68	ns	ns	ns	1	ns	ns	ns	2.75	ns	partial 2-IgSF FREP	FREPJ1
Bg38205	ns	-3.62	-3.69	1.28	2.62	2.29	ns	ns	ns	ns	ns	partial 1-IgSF FREP	new partial FREP
Bg177	ns	ns	3.86	ns	ns	ns	ns	ns	ns	ns	ns	sFReD	
Bg178	ns	ns	2.79	ns	ns	ns	ns	ns	ns	ns	ns	sFReD	
Bg2450	ns	3.1	ns	ns	ns	ns	ns	ns	ns	ns	ns	sFReD	
Bg2494	ns	ns	-5.03	ns	ns	ns	ns	ns	ns	ns	-5.69	sFReD	
Bg12039	-3.93	-3.43	ns	ns	ns	ns	ns	ns	ns	ns	ns	partial sFReD	
Bg16307	ns	4.38	-4.36	ns	ns	ns	ns	ns	ns	4.12	-7.23	sFReD	
Bg16436	ns	1.23	ns	ns	ns	ns	ns	ns	ns	ns	ns	partial sFReD	
Bg17605	ns	ns	-1.68	ns	ns	ns	ns	ns	ns	ns	ns	sFReD	
Bg18336	ns	ns	ns	1.14	ns	ns	ns	ns	ns	ns	ns	sFReD	za_tig_4714
Bg20351	ns	ns	ns	3.46	6.59	2.98	ns	ns	ns	ns	ns	sFReD	BgMFREP4_AY012701-like
Bg25643	ns	1.24	ns	1.64	ns	ns	ns	ns	ns	ns	ns	sFReD	
Bg28385	5.85	5.88	ns	2.55	ns	ns	ns	3.36	3.82	ns	ns	sFReD	
Bg34525	ns	ns	ns	1.6	ns	ns	ns	ns	ns	ns	ns	partial sFReD	
Bg37621	ns	ns	ns	2.83	ns	ns	ns	ns	ns	ns	ns	partial sFReD	
Bg38723	ns	ns	ns	ns	ns	ns	ns	ns	ns	ns	-5.61	sFReD	
Bg40034	4.55	3.82	2.06	ns	1.11	ns	ns	ns	ns	ns	ns	partial sFReD	16_tig_2402_BGLB007591-like
Bg40437	ns	ns	-4.14	ns	ns	ns	ns	ns	ns	ns	-5.16	partial FReM	FReM

**Notes**:

Value in cells represents the value of log2(fold change) or log2(FC). Any up-regulated gene with log2(FC) ≥1 is highlighted in red, and any down-regulated gene with log2(FC) ≤-1 is highlighted in blue.

**0.5d**, **2d**, **8d**, **40d** represent the 4 sampling time points. They include time-matched unexposed controls and exposed snails for valid comparisons.

**ns**: no significant expression difference detected. Gene with PPDE ≥0.95 and FC ≥2 in EBSeq analysis is considered to be differentially expressed in this study.

In the column of “**Type of FReD”**, *B*. *glabrata* FReD proteins were considered “partial” based on the criteria set in this study. “1-IgSF FREP” and “2-IgSF FREP” represent numbers of IgSF domains in a particular FREP.

“**Best hit to published FREP**” in the last column represents the best hit of the protein against published *B*. *glabrata* FREP sequences from available sources [30,32–34]. Any protein sequence identity ≥90% is considered as the same FREP; identity 85~90% is considered as the corresponding FREP “-like”; identity <85% is not assigned to any published FREP. Several FREPs were highlighted in yellow to emphasize in this study.

In the differential expression analysis, SUS unexposed snails were set as baseline for the corresponding time-matched RES unexposed snails at constitutive level; RES unexposed snails were used as baseline for the corresponding time-matched RES snails exposed to *S*. *mansoni*; similarly, SUS unexposed snails were used as baseline for the corresponding time-matched SUS snails exposed to *S*. *mansoni*.

With respect to snails exposed to *S*. *mansoni*, within strain comparisons were made using unexposed snails as a baseline. For RES snails, *S*. *mansoni* miracidia penetrate and transform into mother sporocysts but are typically attacked by host hemocytes and destroyed within 8 days post-exposure (dpe). Many FReD genes (18 FREPs and 7 sFReDs) were responsive to exposure to *S*. *mansoni* in RES snails, all of which were up-regulated, and most were responsive at 0.5–8 days dpe (Table 3).

In comparison, in SUS snails, *S*. *mansoni* miracidia penetrate, transform into mother sporocyst which produce daughter sporocysts which grow and eventually successfully produce massive numbers of cercariae beginning about 5 weeks after infection. Three FREPs and 3 sFReDs were transiently and weakly up-regulated and two FREPs and 4 sFReDs were strongly down-regulated. Down-regulated expression was notably strong later in infection when the snail hosts were producing cercariae. The disparate FREP4 responses of snails of the two strains to *S*. *mansoni* was particularly striking.

With respect to the normalized read counts (S5 Table) and the raw read counts (S6 Table), the expression level of some FREPs and sFReDs are consistently high in SUS snails. For instance, the normalized read counts of FREP3.2 (Bg204) in SUS are high (>1,900 reads) with or without exposure to *S*. *mansoni* during the 4 sampling time points. Similarly, Bg23857 (newly found partial FREP) also has consistent high and stable read counts (250+ reads) in SUS snails regardless of exposure to *S*. *mansoni*. Bg20516, one of the new partial 2-IgSF FREPs found, is expressed in RES strain with average normalized counts of 156 in RES samples, while the average normalized read counts was less than 1 (<1) in SUS strain.

We also directly compared RES and SUS snails exposed to *S*. *mansoni* (S7 Table). Caution in interpretation is required here as we have noted the two strains have quite different baseline representations of FReDs, and they respond so differently to *S*. *mansoni*. Interestingly, the expression pattern noted accentuates the differences we have already noted for the within strain comparisons noted above: RES snails show many up-regulated FReDs especially shortly following exposure and SUS mostly show strong patterns of down-regulation later in the course of infection.

Additionally, the following observations for S4 Table were noted: 1) Compared to FREPs, the read counts numbers of sFReDs in either strain were modest and did not respond as dramatically to *S*. *mansoni*; 2) The normalized average constitutive read counts among FREPs from SUS snails range from 0–3,500 with FREP3.2 being the most expressed FREP (>3,500), and from 0–3,366 for RES snails with FREP2 being the highest expressed (for one snail was >6,400). A greater variety of FREPs were expressed at high constitutive levels and post-exposure levels for RES than SUS snails (with cut-off >100 normalized reads counts) in S5 Fig; 3) The overall changes in read counts were modest for FREPs from SUS snails exposed to *S*. *mansoni*, even for the constitutively highly expressed FREP3.2. By contrast, RES snails had much more pronounced responses especially for FREP2, 3.1, 3.2, 4, J1, J5, 14, and one of the new FREPs (Bg20516*); 4) Of the 4 most abundant FREPs in RES snails responding to *S*. *mansoni* (2, 3.1, 3.2 and 4), only read counts for one of them, FREP 3.2, were as common in SUS snails. Although lack of a fully annotated sequence prevented a complete expression analysis for the presumptive FREP3.3 locus, our preliminary read count values for it mirror those of FREP3.1 and 3.2 in showing a strong up-regulation in RES but not SUS snails following exposure to *S*. *mansoni*.

### Variant FREP transcripts from RES and SUS snails

**Fig 8:**
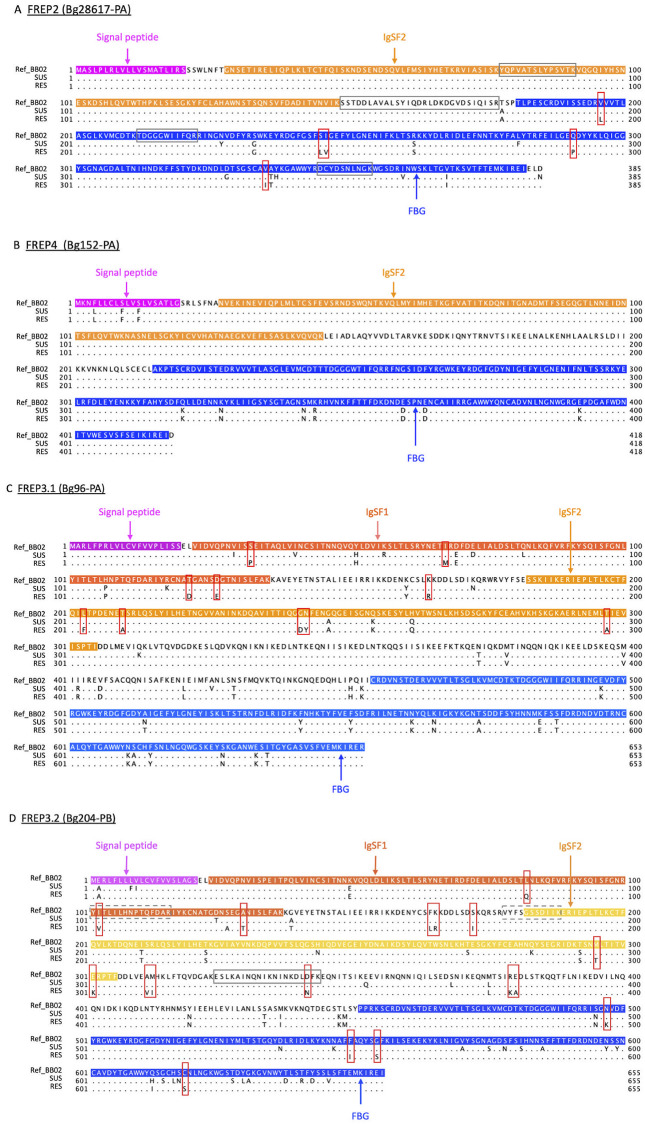
Single nucleotide variant (SNV) analysis in *B*. *glabrata* FREPs between SUS and RES with RNA-Seq sequences. SNV analysis of (**A**) FREP2 (Bg28617-PA); (**B)** FREP4 (Bg125-PA); (**C)** FREP3.1 (Bg96-PA); and (**D)** FREP3.2 (Bg204-PB). “**Ref_BB02**”, “**SUS**”, and “**RES**” are protein sequences of corresponding FREPs; in which RES and SUS are regenerated based on SNVs in their transcriptome data with BB02 as reference. Four featured sequences of “Signal peptide”, “IgSF1”, “IgSF2” and “FBG” are highlighted in colors. Each SNVs in RES from the two susceptible strains of BB02 and SUS are boxed in red. Any absolute matched sequences to identified peptides from FREP2, FREP3.2 of the plasma of SUS or RES from the pull-down experiments by the Li et al. [47] are framed in gray boxes with solid line. Sequences in FREP3.2 absolutely matched to the two RES-specific peptides from the pull-down experiments by Wu et al. [49] are framed in gray boxes with dash line.

Protein sequences for four FREPs indicated in previous literature to be pivotal to the *B*. *glabrata* response to trematodes, FREPs 2, 3.1, 3.2 and 4, were generated from available transcripts (Fig 8) and aligned with corresponding proteins from the BB02 genome. Note that BB02 snails are also susceptible to schistosome infection. The SNV analysis focused on coding regions of these FREP genes and used cut-off scores of quantity >10 and quality scores of filtering variants of >20. The single nucleotide variants (SNVs) shown are only those that result in actual coding change in amino acids, at the positions shown in Fig 8. Other SNVs were identified in Fig 8 but did not differentiate RES from SUS snails, so are not discussed further.

For FREP2 (Fig 8A), all SNVs unique to RES snails were in the FBG domain. For FREP 3.1 (Fig 8C), RES-specific SNVs were found in both IgSF1 and IgSF2 domains. For FREP 3.2 (Fig 8D), one of the most common FREPs in both SUS and RES snails, RES-specific SNVs were found in IgSF1, IgSF2, ICR and FBG domains. The RES-specific SNV at position #102 aa (within the IgSF1 domain, Fig 8D) overlaps with the peptide (#101-115aa: YITLILHNPTQFDAR) from the RES-specific sporocyst-binding protein identified by Wu et al. [49]. Although FREP 3.1 and FREP 3.2 are similar, there are no SNVs in common between them (Fig 8C and 8D). Interestingly, no SNVs were found in FREP4 (Fig 8B).

Some amino acid changes due to SNVs between SUS and RES strains result in changes of hydropathy score, charge, and biochemical properties [93,94] (S8 Table). For instance, at position 245 in the FREP2 protein sequence, a serine (S) in both BB02 and SUS is replaced by a leucine (L) in RES snails. This amino acid change increases the hydropathy score from -0.8 (S) to 3.8 (L). More details are found in S8 Table. As we pieced together FREP sequences from transcriptomic data, our SNV calls will benefit from verification from genomic sequence data for SUS and RES snails.

Additionally, four absolute matches with the FREP peptide sequences identified by Li et al. [47] were found in FREP2 (Fig 8A). No SNVs were located among these four matched sequences. Similarly, one absolute match with the FREP peptide sequence was found in FREP3.2, and one SNV was located in this matched sequence (Fig 8D).

## Discussion

The freshwater gastropod *B*. *glabrata* and the larval stages of the human-infecting digenetic trematode *S*. *mansoni* together comprise the most prominent model system for exploring snail-trematode interactions. Given that over 18,000 species of digenetic trematodes exist [95], almost all of which require a molluscan intermediate host to complete their life cycles, it is important to gain a deeper understanding of their associations. Proteins containing FBG domains called FReDs, particularly when associated with IgSF domains as in FREPs, have become the most studied group of *B*. *glabrata* molecules, particularly in the context of their responsiveness to trematode infection and involvement in resistance. Many questions remain with respect to FReD biology, including overviews of their genomic representation, domain structures, evolutionary relationships, responsiveness to infection, and their variability among strains of *B*. *glabrata* differing in schistosome susceptibility. We add to our knowledge of FReDs by addressing these issues here.

We found 73 FBG domains encoded in the BB02 *B*. *glabrata* genome using our InterProScan search: a single FReM associated with an EGF-like domain, 39 associated with one or two IgSF domains to comprise the FREPs, and 33 comprised only of an FBG domain and designated as sFReDs.

In searching out the IgSF domains so prominently found in FREPs, customized new HMM search profiles were used. Such search profiles are justified by the fact that IgSF domain sequences are highly diversified in both vertebrates [96,97] and in invertebrates like *B*. *glabrata* [35]. Furthermore, records of vertebrate IgSF sequences predominate (as of February 2020, nearly 80% of the 434 thousand entries for metazoan immunoglobulin-like folds) relative to invertebrate IgSF sequences.

The customized new HMM search allowed us to identify more *B*. *glabrata* IgSF domains than did a conventional search (Table 1 and S2 Table). Consequently, the expanded number of 39 FREPs noted above includes one complete and six partial, previously undocumented new FREP-encoding genes, all of which lack signal peptides that might yet be located with more complete genome assemblies. The automated IgSF and FBG identification workflow presented in Fig 2 is in effect a “FREP finder,” and should facilitate discovery of variable immunoglobulin and lectin domain containing molecules (VIgLs) in other molluscs or invertebrates, at proteome or transcriptome level.

Interestingly, only 2 of the 38 *B*. *glabrata* IgSF HMM profiles we generated are associated with FBG domains to form FREPs. Some of the remaining identified IgSF domains found on isolated contigs may also associate with FBG domains but this too awaits improved genome assemblies.

With respect to unusual features of some of the FReDs examined, one contains an accociation of FREP1 with a PAN/Apple domain, previously discovered in *B*. *glabrata* [32]. PAN/Apple domains are found from proteobacteria to mammals and function in protein-protein or protein-carbohydrate interactions [98–100]. In molluscs, they are known from 4 *Crassostrea gigas* genes [101], and from some *Lottia gigantea* FReDs [56]. FREP1 is remarkable as the only 1-IgSF FREP to possess an IgSF1 instead of an IgSF2 domain. FREP1 is not expressed significantly in RES or SUS snails exposed to *S*. *mansoni*, so it may function in some other unknown context.

Another unusual feature revealed by the genome-wide domain predictions for FREPs pertains to the ICR region, which is usually rather variable and short in length (14–133 aa) but was found to be >100aa in length (and up to 320aa long) in 15 of the 39 complete and partial FREPs. Coiled-coils are prominent within ICR regions and may play a role in multimerization of FREPs [53]. InterProScan scanning results showed that coiled coils 35 aa in length occur in the ICR of FREP4 but were not identified in the ICR of FREP2, in line with previous observations that FREP4 forms multimers, while FREP2 does not [50].

We found the two IgSF domains in 2-IgSF FREPs, IgSF1 and IgSF2, cluster separately from all non-FREP IgSF (branches in gray, Fig 7A) known from *B*. *glabrata*, and can also be differentiated from one another (Fig 7B), as also noted for FREP 2, 3, 4, 7 by Zhang et al. [35]. Fig 7B also suggests that the IgSF2 domain in 1-IgSF FREPs like FREP2 and 4 are divergent from the IgSF2 domains found in 2-IgSF FREPs which show a pattern of diversification from a common source. A similar pattern was found for the IgSF1 domains. FREP1 with its divergent IgSF1 domain grouped separately from the rest of the IgSF1 domains, all of which are found in 2-IgSF FREPs. The latter IgSF1 domains also show a pattern of diversification from a common source. Along with the results from Fig 6C, the pattern suggests that the two IgSF domains have diverged in parallel.

The plots of distributions of protein sequence diversity (Fig 5) suggest for FREPs that the IgSF2 domain is significantly more variable than the IgSF1 domain (*p* = 7.82^−22^). They also show that the FBG domains associated with FREPs are relatively conserved, compared both to the IgSF domains [30], and to FBG domains found in sFReDs. The FBG domain in FREPs may be under selection to conserve an essential function whereas the greater diversity of FBG domains in sFReDs may reflect a broader range of functional roles, or a broader collective pathogen carbohydrate recognition capacity. The role of sFReDs in defense in *B*. *glabrata*, however, remains somewhat conjectural. Parts of two sFReDs were found by [49] to bind *S*. *mansoni* sporocysts. Relatively few of the sFReDs we recovered had homology to the sporocyst-binding peptides identified by Wu et al. (2017) [49]. Furthermore, as indicated in Table 3, we saw but little evidence of sFReDs responding to trematode infection. Four complete sFReDs (Bg18336, Bg20351, Bg25643, and Bg28385) were upregulated in RES strains at 12 hours post-exposure to *S*. *mansoni*; two partial sFReDs (Bg34525 and Bg37621) were also upregulated in RES strains (Table 3). These six sFReDs deserve additional study in anti-trematode contexts.

One of the goals of this study was to investigate how the FReDs of SUS and RES snails differ with respect to their expression before and after exposure to *S*. *mansoni*. As our first approach, several peptide sequences from the proteomic study of Wu et al. [49] were matched to the FREP sequences identified in the current study (Fig 4), and in some cases strain differences were noted among the sporocyst-bound plasma peptides identified [49]. For instance, 3 peptide sequences fully matched on some portion of RES FREP3.2, one each in IgSF1, IgSF2 and FBG domains. Only one peptide sequence matched SUS FREP3.2. Additionally, only the first RES-specific peptide in IgSF1(#101-115aa: YITLILHNPTQFDAR) overlaps with one RES-specific SNV (red box highlighted, at #102aa) in FREP3.2 (Fig 8D), which may be relevant to the role of FREP3.2 in resistant to *S*. *mansoni*. There were 11 peptide sequences from SUS snails that fully matched portions of a new partial FREP (Bg38205), representing IgSF2, long ICR and FBG regions of the molecule. In contrast, only two peptide sequences from RES snails matched sequences of this FREP, both in the FBG domain. From our expression studies, this same FREP shows higher constitutive expression in unexposed SUS relative to RES snails, but following exposure to *S*. *mansoni*, it is up-regulated in RES but not SUS snails (Table 3 and S4 Table). It is one of several FREPs we discuss worthy of further study, in this case because it is clearly capable of binding to sporocysts.

Our second approach to looking at strain differences in responses to *S*. *mansoni* was to interrogate our RNA-Seq data with respect to the comprehensive list of FReDs we gleaned from the *B*. *glabrata* genome and from assembled transcripts (Table 3 and S4 Table). With respect to constitutive levels of FReD expression, the overall comparison between SUS and RES snails is mixed. For instance, RES snails do not have dramatically higher resting levels of FREPs than SUS snails. This is somewhat in contrast to our recent study of GTPase IMAP family members (GIMAPs) showing that over 20 members of this gene family are constitutively expressed at significantly higher levels in RES than in SUS snails, in both juvenile and adult stages [84]. Although normalized read counts may not provide the best indicator of true relative abundance among various FReD transcripts, it is clear that some FREPs are expressed at higher constitutive levels than others. Especially noteworthy is FREP 3.2 in SUS snails with a consistently high read count level across all samples.

For FREPs in particular, we found a greater variety were expressed in RES than SUS snails both before and after *S*. *mansoni* exposure (S4 Fig). This observation is consistent with a transcriptome study on multistrain compatibility comparisons in *B*. *glabrata* [34]. In their study, 4 strains of *B*. *glabrata* snails were compared, BgVEN, BgGUA, BgBRE and BgBAR, of which BgBAR is the least susceptible to *S*. *mansoni*. They used only unexposed snails and found BgBAR had the highest level of FREPs expression among their four strains [34].

What we found to be particularly remarkable is how responsive the RES FREP repertoire was relative to SUS snails following *S*. *mansoni* exposure, especially during the 0.5–8 dpe interval when RES snails are actively rejecting infection (Table 3 and S4 Table). FREPs 2 and 4 are noteworthy for the extent of their early up-regulation in RES snails in comparison to SUS snails which are either unresponsive or show sharp-down-regulation, confirming the previous qPCR results of Hertel et al. (2005) [41]. We note that previous studies based on microarray results [39] found FREP4 in particular to be more responsive in SUS snails, so much so as to be a marker of infection, but our RNA-Seq studies using different methods and snail groups did not confirm this.

Both FREP3.1 and FREP3.2 (and tentatively FREP3.3) were both also strongly up-regulated in RES snails shortly following exposure to infection, lending support to a role for FREP3.2 in the resistance response of snails to trematode parasites [38,40,47]. SUS snails consistently showed high levels of expression of FREP 3.2, but neither this FREP nor any other showed pronounced up-regulation following exposure to *S*. *mansoni*, except FREP2. This FREP showed upregulation only in snails actively shedding cercariae, but most other FReDs showed no response or down-regulation in shedding snails. A transcriptome study of Kenyan field-derived *Biomphalaria pfeifferi*, a snail species highly susceptible to *S*. *mansoni*, found that FREP2 was consistently up-regulated during infection, FREPs 5, 7,14 were upregulated in early and shedding stages [102], while FREP12 was consistently down-regulated in *B*. *pfeifferi* in early infections (1- and 3-day post-infection). FREP3 was minimally responsive in this Kenyan *B*. *pfeifferi* study. It was modestly up-regulated only at 1d, in the snail with fewest *S*. *mansoni* reads, and the FREP4 homolog was not expressed in *B*. *pfeifferi* during the infection.

Some other comparisons with past studies are as follows: 1) for FREP5, we noted no changes in its expression whereas down-regulation in SUS snails after 4 dpe to *S*. *mansoni* was noted in a microarray study [39]; 2) FREP7 was noted to be modestly upregulated in RES snails and unresponsive in SUS snails whereas [41] found it to be highly upregulated in SUS snails by qPCR, and Adema et al. (2010) found its response to bacterial exposure to be mixed; 3) for FREP14, we noted modest up-regulation in RES snails following exposure to *S*. *mansoni* although no significant responses were observed using qPCR in expression for either RES or SUS snails after exposure to *S*. *mansoni* [43].

To summarize this aspect of our study, RES snails showed strong upregulated FREP responses following exposure to *S*. *mansoni* whereas SUS snails did not. Some FREPs worthy of additional consideration include FREPs A, J1 and J2 as well as Bg38205 and Bg20516. In addition, four FREPs that persistently turn up of being of interest in previous studies [20], [26,29,31,35,38–44,47,49] were again prominent players: FREPs 2, 4 and FREPs 3.1 and 3.2. These FREPs are responsive to infection and expressed at relatively high levels. Also, relative to FREPs, sFReDs seem to be expressed at lower levels and did not show conspicuous patterns of response though Bg20351, Bg28385 and Bg37621 from RES snails should not be discounted and some modest changes in sFReDs were noted in SUS snails.

As the third component of our examination of FReD responses, given the relative abundance and prominence of FREPs 2, 3.1, 3.2 and 4 responses in RES relative to SUS snails, we looked for interstrain amino acid coding differences among these FREPs (Fig 8). No interstrain amino acid substitutions occurred for FREP4, but substitutions were found for FREPs 2, 3.1 and 3.2. For FREP2, all inter-strain coding differences were found in the FBG domain whereas for FREP3.2 coding differences were found in IgSF1, IgSF2 and FBG domains. Interestingly, FREP3.1 and FREP3.2 do not share any SNVs. Most SNVs in FREP3.1 are in IgSF1 and 2 domains, and SNVs in FREP3.2 are mainly in the IgSF1 and FBG domains. Although we have been unable to do a full analysis on FREP3.3, preliminary sequence analysis suggests this FREP also shows interstrain differences. Although these results need to be confirmed with genomic sequence data for these two snail strains, they suggest that along with considerable differences in expression profiles, resistant BS-90 and susceptible M line snails have different alleles at these key loci. Accordingly, these results fall in line with the interaction model proposed by Li et al. [47] from the specific standpoint that for both the various forms of FREP 3 (3.1, 3.2 and likely 3.3) and for FREP2, it was postulated that the forms involved in responding to *S*. *mansoni* sporocysts are different between SUS and RES snails.

We emphasize we are not claiming that differences in FREP expression or allelic composition represent *the sole* basis of resistance in *B*. *glabrata* to *S*. *mansoni* as several other hypotheses are in play [17,65,103]. Our genome-wide and inter-strain comparative approach focused on a group of molecules of long-standing interest to us, *B*. *glabrata* FREPs and sFReDs, and is the first to provide a comprehensive overview of transcriptional activity involving molecules with identified amino acid coding differences between schistosome-susceptible and -resistant *B*. *glabrata* strains. In developing models for how resistance is manifested at the molecular level, both expression changes and allelic differences among snails in key loci clearly need to be considered.

Also, with respect to applying our results to real world situations where *S*. *mansoni* is being transmitted, we need to determine what FREP loci are present in local *Biomphalaria* sp. vector populations, whether allelic versions of key FREPs may exist in such populations as well, and monitor what alleles are present in individual snails that are able to reject *S*. *mansoni* infections or not. It will be of particular interest to learn if certain FREP genotypes might vary in time relative to extant infectivity genes in *S*. *mansoni* in accordance with matching alleles models of compatibility [104–106] or whether a different, more generalized anti-trematode strategy, possibly based on FREPs as well, also exists in snail populations experiencing heavy burdens of parasitism from a variety of trematode species [107].

## Supporting information

S1 Table Summary of sequencing samples information in RNA-Seq study. Notes: **nd**: not done, *: The 2 dpe unexposed group was used for the 8 dpe comparison as well, because they did not differ in size. **dpe**: day post-exposure to *S*. *mansoni*. **Unexposed**: control snails not exposed to *S*. *mansoni* and were set to be time-matched to a corresponding exposed group that was.
(XLSX)

S2 Table Manual checking summary of 4 FReD genes confirmed as FREPs. (XLSX)

S3 Table Summary of predicted 95 FReD proteins in *B*. *glabrata* BB02 strain in details. Notes: 1. In the column of “**ID in text**”, multiple proteins encoded by the same gene due to alternative splicing were differentiated by different version of protein, such as “-PA”, “-PB”, “-PC”, “-PD” or “-PE” suffix following the gene ID. 2. In the column of “**NCBI Annotation**”, “**—**” represents no available annotation for a particular protein; 3. In the column of “**Type of FReD”**, each particular FReD was considered as complete or partial based on the criteria set in this study above; “1-IgSF FREP” and “2-IgSF FREP” represent the number of IgSF domains present in a particular FREP; 4. “**Best hit to published FREP**” represent the best hit of individual protein against published *B*. *glabrata* FREP sequences from available sources [30,32–34]: best hit of protein sequence identity ≥90% is considered as the same FREP; identity 85~90% is considered as “-like” of the corresponding FREP; identity <85% is not assigned to any known FREP. Several FREPs were highlighted in yellow to emphasize in this study. 5. In the column of "**IgSF2 length (aa)**", texts in red indicate truncated domains, which are possible to be extended to complete domains with improved assembly or annotation; text in blue indicates a residue of IgSF domain, which is not likely to be extended to a complete domain due to the location between two complete domains; 6. Asterisk marked 4 FREPs IDs in the first two columns were extended sequences after manual checking. 7. The highlighted gene "**Bg6034**" is a partial 2-IgSF FREP with disrupted architecture: the partial FBG domain was not located at C terminal but inserted into the IgSF2 domain, which was broken into two parts. From the VectorBase genome browser view, there is a gene with 7 exons spans over two contigs APKA01042331.1 (exon 4–7) and APKA01042332.1 (exon 1–3). This unusual gene sequence is most likely a genome assembly error; no matching sequences of transcripts were found among the transcripts from 12 different *B*. *glabrata* organs [32].
(XLSX)

S4 Table Summary of pairwise blast identity of IgSF, FBG domains in complete FReDs (FREP and sFReD) Note: Mann-Whitney U test P values were listed below diagonal, statistical test values listed above diagonal. (XLSX)

S5 Table Normalized average read counts for each group in RES and SUS with or without exposure to *S*. *mansoni* Notes: 1. Values in cells represent the average normalized read counts for each group at different sampling time points. 2. “**0.5dpe, 2dpe, 8dpe, 40dpe**” represent the 4 sampling time points. They include time-matched unexposed controls and exposed snails for valid comparisons. 3. In the column of “**Type of FReD**”, *B*. *glabrata* FReD proteins were considered “partial” based on the criteria set in this study. “1-IgSF FREP” and “2-IgSF FREP” represent numbers of IgSF domains in a particular FREP. 4. In the column of “**ID used in text**”, IDs highlighted in gray represent the differentially expressed (DE) FReD genes in any comparison applied. 5. In the column of “**Best hit to published FREP**”, the best hit of the protein against published *B*. *glabrata* FREP sequences from available sources [30,32–34] were listed. Any protein sequence identity ≥90% is considered as the same FREP; identity 85~90% is considered as the corresponding FREP “-like”; identity <85% is not assigned to any published FREP. Several FREPs were highlighted in yellow to emphasize in this study. 6. In the differential expression analysis, SUS unexposed snails were set as baseline for the corresponding time-matched RES unexposed snails at constitutive level; RES unexposed snails were used as baseline for the corresponding time-matched RES snails exposed to *S*. *mansoni*; similarly, SUS unexposed snails were used as baseline for the corresponding time-matched SUS snails exposed to *S*. *mansoni*.
(XLSX)

S6 Table Raw read counts for each replicate snail sample in RES and SUS with or without exposure to *S*. *mansoni* Notes: 1. Values in cells represent raw read counts for each snail sample/each replicate at different sampling time points. 2. “**R1, R2, R3, R4, R5, R6**” represent the number of replicates for each particular group (unexposed or exposed to *S*. *mansoni*) 3. “**0.5dpe, 2dpe, 8dpe, 40dpe**” represent the 4 sampling time points. They include time-matched unexposed controls and exposed snails for valid comparisons. 4. In the column of “**Type of FReD**”, *B*. *glabrata* FReD proteins were considered “partial” based on the criteria set in this study. “1-IgSF FREP” and “2-IgSF FREP” represent numbers of IgSF domains in a particular FREP. 5. In the column of “**ID used in text**”, IDs highlighted in gray represent the differentially expressed (DE) FReD genes in any comparison applied. 6. In the column of “**Best hit to published FREP**”, the best hit of the protein against published *B*. *glabrata* FREP sequences from available sources [30,32–34] were listed. Any protein sequence identity ≥90% is considered as the same FREP; identity 85~90% is considered as the corresponding FREP “-like”; identity <85% is not assigned to any published FREP. Several FREPs were highlighted in yellow to emphasize in this study. 7. In the differential expression analysis, SUS unexposed snails were set as baseline for the corresponding time-matched RES unexposed snails at constitutive level; RES unexposed snails were used as baseline for the corresponding time-matched RES snails exposed to *S*. *mansoni*; similarly, SUS unexposed snails were used as baseline for the corresponding time-matched SUS snails exposed to *S*. *mansoni*.
(XLSX)

S7 Table Differential expression analysis of *B*. *glabrata* FReD genes between RES-exposed and SUS-exposed to *S*. *mansoni* at different time points Notes: 1. Value in cells represents the value of log2(fold change) or log2(FC). Any up-regulated gene with log2(FC) ≥1 is highlighted in red, and any down-regulated gene with log2(FC) ≤-1 is highlighted in blue. 2. **“0.5dpe**, **2dpe**, **8dpe**, **40dpe”** represent the 4 sampling time points. They include time-matched unexposed controls and exposed snails for valid comparisons. 3. **ns**: no significant expression difference detected. Gene with PPDE ≥0.95 and FC ≥2 in EBSeq analysis is considered to be differentially expressed in this study. 4. In the column of “**Type of FReD”**, *B*. *glabrata* FReD proteins were considered “partial” based on the criteria set in this study. “1-IgSF FREP” and “2-IgSF FREP” represent numbers of IgSF domains in a particular FREP. 5. “**Best hit to published FREP**” in the last column represents the best hit of the protein against published *B*. *glabrata* FREP sequences from available sources [30,32–34]. Any protein sequence identity ≥90% is considered as the same FREP; identity 85~90% is considered as the corresponding FREP “-like”; identity <85% is not assigned to any published FREP. Several FREPs were highlighted in yellow to emphasize in this study. 6. In the differential expression analysis, SUS unexposed snails were set as baseline for the corresponding time-matched RES unexposed snails at constitutive level; RES unexposed snails were used as baseline for the corresponding time-matched RES snails exposed to *S*. *mansoni*; similarly, SUS unexposed snails were used as baseline for the corresponding time-matched SUS snails exposed to *S*. *mansoni*.
(XLSX)

S8 Table Summary of amino acid change in coding region of interested FREPs between *B*. *glabrata* susceptible strains (BB02 and SUS) and resistant strain (RES) Notes: 1. The “**Hydropathy Score**” is a quantitative analysis of the degree of hydrophobicity or hydrophilicity of amino acids of a protein [93]. 2. **pKa** is the negative of the logarithm of the dissociation constant for the -COOH group; **pKb** is the negative of the logarithm of the dissociation constant for the -NH_3_ group; **pKx** is the negative of the logarithm of the dissociation constant for any other group in the molecule; **pI** is the pH at the isoelectric point [92].
(XLSX)

S1 Fig Diagram of predicted FREP genes with new HMM profiles search approach in *B*. *glabrata*. (TIFF)

S2 Fig Gene tree and representative protein domain architecture of 73 *B*. *glabrata* FReDs based on FBG domain only. **A**. Maximum likelihood tree with 1000 bootstrap test was constructed based on FBG domain sequences of 73 FReD genes in *B*. *glabrata*. Nodes with bootstrap support of 75 or higher are marked with black squares. The gene IDs of the 73 FReDs were followed with number of IgSF domains and the best hit with any published FREP genes (BLASTp hit with at least 150 aa aligned with ≥90% identity was considered as the same FREP; or considered a “FREP-like” gene if identity was 85% ~ 90%; identity <85% was not assigned to any published FREP). Any FReD genes with best hit to FREPs from the genome paper [32] are labeled with a red dot at the corresponding gene ID. Complete FREP or sFReD genes fitting the criteria in this study were highlighted in blue text. **B**. Domain architectures were predicted using InterProScan and lineage specific HMM models. “**Peptide sequences**” represent multiple peptide sequences extracted from the proteomics study [49]. Colored small horizontal bars above or below some protein domain structures highlight the matched location of peptide sequences with binding affinity to *S*. *mansoni* sporocyst membrane-enriched and larval transformation proteins, from either schistosome-susceptible NMRI strain (SUS in green) or -resistant BS-90 strain (RES in red) of *B*. *glabrata*. The dashed gray line was manually added to distinguish most “sFReD clades” from “FREP clades”.
(TIFF)

S3 Fig Genomic locations of IgSF and FBG domains containing genes in *B*. *glabrata* BB02 strain. (TIFF)

S4 Fig Gene tree, representative protein domain architecture and gene structure of 73 *B*. *glabrata* FReDs. **A**. Maximum likelihood tree with 1000 bootstrap test was constructed using full length representative protein sequences of 73 FReD genes in *B*. *glabrata*. Nodes with bootstrap support of 75 or higher are marked with black squares. The gene IDs of the 73 FReDs were followed with number of IgSF domains and the best hit with any published FREP genes (BLASTp hit with at least 150 aa aligned with ≥90% identity was considered as the same FREP; or considered a “FREP-like” gene if identity was 85% ~ 90%; identity <85% was not assigned to any published FREP). Any FReD genes with best hit to FREPs from the genome paper [32] are labeled with a red dot at the corresponding gene ID. Complete FREP or sFReD genes fitting the criteria in this study were highlighted in blue text. **B**. Domain architectures were predicted using InterProScan and lineage specific HMM models. “**Peptide sequences**” represent multiple peptide sequences extracted from the proteomics study [49]. Colored small horizontal bars above or below some protein domain structures highlight the matched location of peptide sequences with binding affinity to *S*. *mansoni* sporocyst membrane-enriched and larval transformation proteins, from either schistosome-susceptible NMRI strain (SUS in green) or -resistant BS-90 strain (RES in red) of *B*. *glabrata*. The dashed gray line was manually added to distinguish most “sFReD clades” from “FREP clades”. **C**. Gene structures of the 73 *B*. *glabrata* FReD genes.
(TIFF)

S5 Fig Comparisons of numbers of FREPs expressed in RES and SUS before and after *S*. *mansoni* exposure. With the cut-off >100 normalized read counts, number of expressed FREPs in RES or SUS strains with or without exposure to *S*. *mansoni* were summarized.
(TIFF)
