# Supplementary figures and images for: Genomic and transcriptional analysis of genes containing fibrinogen and IgSF domains in the schistosome vector Biomphalaria glabrata, with emphasis on the differential responses of snails susceptible or resistant to Schistosoma mansoni
Source: PLoS Negl Trop Dis. 2020 Oct 14;14(10):e0008780. doi: 10.1371/journal.pntd.0008780 (PMC7588048; doi:10.1371/journal.pntd.0008780)

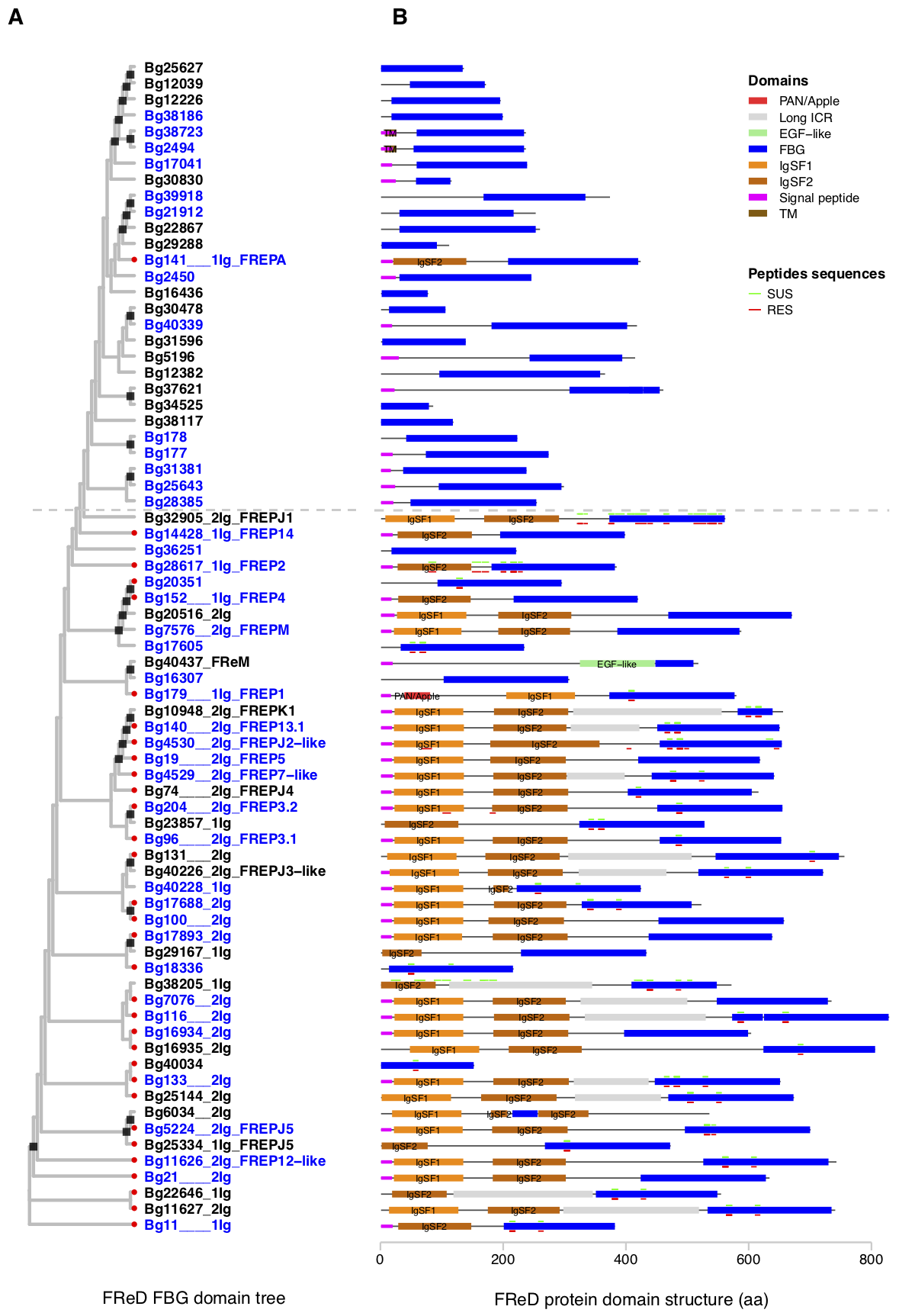

Supplement: S2 Fig — A. Maximum likelihood tree with 1000 bootstrap test was constructed based on FBG domain sequences of 73 FReD genes in B. glabrata. Nodes with bootstrap support of 75 or higher are marked with black squares. The gene IDs of the 73 FReDs were followed with number of IgSF domains and the best hit with any published FREP genes (BLASTp hit with at least 150 aa aligned with ≥90% identity was considered as the same FREP; or considered a “FREP-like” gene if identity was 85% ~ 90%; identity <85% was not assigned to any published FREP). Any FReD genes with best hit to FREPs from the genome paper [32] are labeled with a red dot at the corresponding gene ID. Complete FREP or sFReD genes fitting the criteria in this study were highlighted in blue text. B. Domain architectures were predicted using InterProScan and lineage specific HMM models. “Peptide sequences” represent multiple peptide sequences extracted from the proteomics study [49]. Colored small horizontal bars above or below some protein domain structures highlight the matched location of peptide sequences with binding affinity to S. mansoni sporocyst membrane-enriched and larval transformation proteins, from either schistosome-susceptible NMRI strain (SUS in green) or -resistant BS-90 strain (RES in red) of B. glabrata. The dashed gray line was manually added to distinguish most “sFReD clades” from “FREP clades”. (TIFF) [file pntd.0008780.s010.tiff]

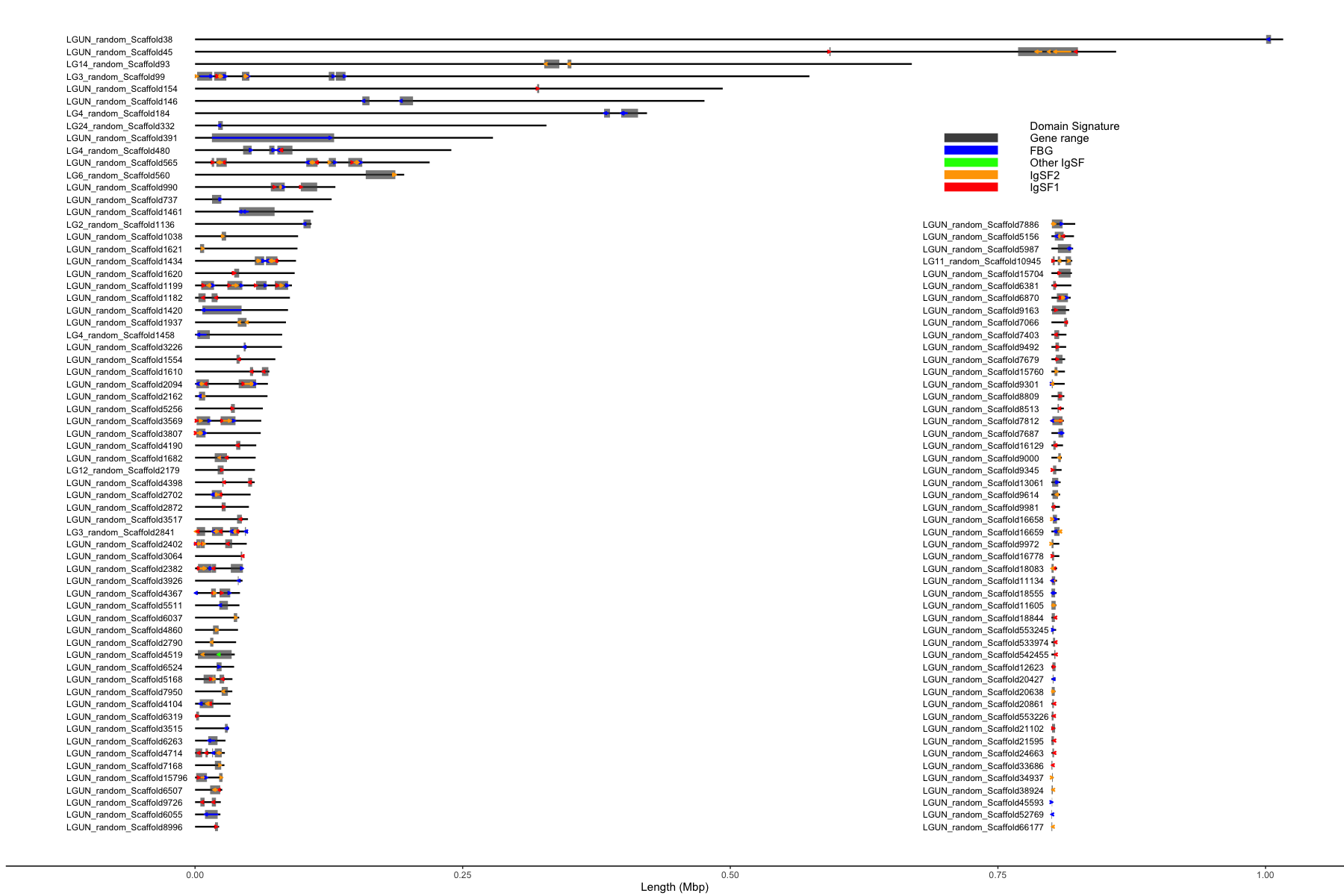

Supplement: S3 Fig — (TIFF) [file pntd.0008780.s011.tiff]

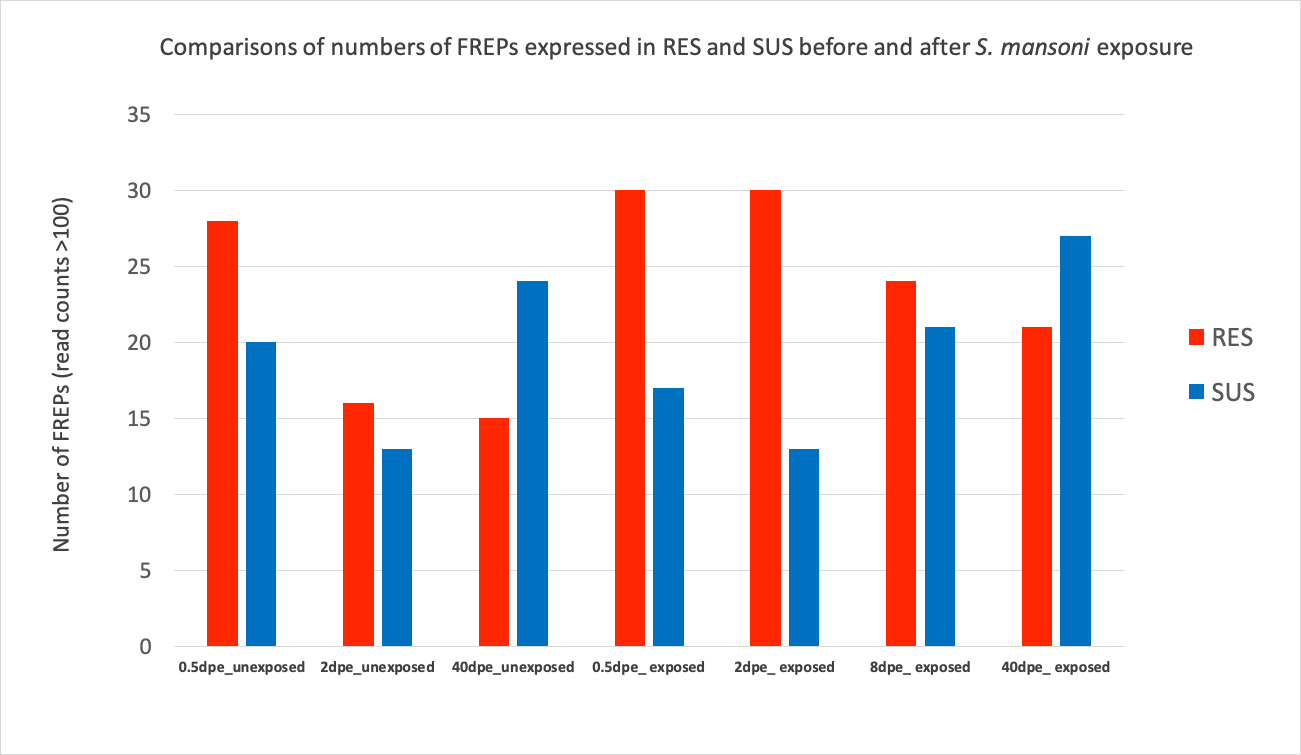

Supplement: S5 Fig — With the cut-off >100 normalized read counts, number of expressed FREPs in RES or SUS strains with or without exposure to S. mansoni were summarized. (TIFF) [file pntd.0008780.s013.tiff]
